# Resistance to host antimicrobial peptides mediates resilience of gut commensals during infection and aging in *Drosophila*

**DOI:** 10.1073/pnas.2305649120

**Published:** 2023-08-28

**Authors:** Aranzazu Arias-Rojas, Dagmar Frahm, Robert Hurwitz, Volker Brinkmann, Igor Iatsenko

**Affiliations:** ^a^Research group Genetics of host–microbe interactions, Max Planck Institute for Infection Biology, Berlin 10117, Germany; ^b^Department of Biology, Chemistry, and Pharmacy, Freie Universität Berlin, Berlin 14195, Germany; ^c^Protein Purification Core Facility, Max Planck Institute for Infection Biology, Berlin 10117, Germany; ^d^Microscopy Core Facility, Max Planck Institute for Infection Biology, Berlin 10117, Germany

**Keywords:** *Drosophila*, microbiota, infection, antimicrobial peptides, *Lactobacillus*

## Abstract

Resilience to short-term perturbations, like inflammation, is a fundamental feature of microbiota, yet the underlying mechanisms of microbiota resilience are incompletely understood. Here, we show that *Lactiplantibacillus plantarum*, a major *Drosophila* commensal, stably colonizes the fruit fly gut during infection and is resistant to *Drosophila* antimicrobial peptides (AMPs). By transposon screening, we identified *L. plantarum* mutants sensitive to AMPs. These mutants were impaired in peptidoglycan O-acetylation or teichoic acid D-alanylation, resulting in increased negative cell surface charge and higher affinity to cationic AMPs. AMP-sensitive mutants were cleared from the gut after infection and aging-induced gut inflammation in wild-type, but not in AMP-deficient flies, suggesting that resistance to host AMPs is essential for commensal resilience in an inflamed gut environment. Thus, our work reveals that in addition to the host immune tolerance to the microbiota, commensal-encoded resilience mechanisms are necessary to maintain the stable association between host and microbiota during inflammation.

## Significance

Intestinal microbial communities are often exposed to host immune effectors triggered by the pathogens during intestinal infection. How commensals stably persist in the gut during infection-induced immune response remains mostly unexplored. Here, we exploited the genetic tractability of the fruit fly *Drosophila melanogaster* and its symbiont *Lactiplantibacillus plantarum* to investigate the mechanism of resilience to inflammation. We found that *L. plantarum* resistance to host antimicrobial peptides (AMPs) mediated by cell wall modifications is essential for resilience in an inflamed gut environment. Our study not only characterizes mechanisms of bacterial resistance to AMPs and symbiont resilience mechanisms to inflammation but also demonstrates that AMP resistance historically associated with virulence of pathogens is also essential to maintain stable microbiota–host interactions.

Gut-associated microbial communities stably colonize the host over the lifetime of an individual despite constant exposure to perturbations which often transiently change microbial community function and composition (1). Over time, the community can revert to the original state as the disturbance passes, thus exhibiting resilience (2, 3). However, when resilience mechanisms fail, perturbations may lead to the establishment of dysbiosis, a disease-associated state of the microbiota, which negatively influences the health of the host (4). Hence, understanding the molecular mechanisms of microbiota resilience is important for maintaining the health of the host. Some perturbations to which the intestinal microbiota is often exposed to are nonspecific inflammatory responses induced by infection. Immune defense mechanisms non-specifically target conserved molecular patterns present in both pathogens and commensals, raising the question of how commensals survive such inflammatory responses and can stably colonize the host. Recent studies have started addressing this question. For instance, human commensal Bacteroidetes modify their lipopolysaccharide structure, leading to increased resistance to host antimicrobial peptides (AMPs) and persistence in the gut during inflammation (5). Another perturbation to which commensals are exposed to during infection is an inflammation-induced iron limitation. *Bacteroides thetaiotaomicron* survives such iron-limiting conditions by utilizing siderophores produced by members of the *Enterobacteriaceae* family to acquire iron (6). This xenosiderophore utilization suggests a crucial role for interspecies iron metabolism in mediating commensal resilience during gut inflammation. However, despite the latest efforts, our understanding of the molecular mechanisms that underlie microbiota resilience during infection remains limited.

One approach to explore microbiota resilience mechanisms toward immune defenses is to use in vivo model systems amenable to genetic manipulation of both host and microbiota members. The fruit fly *Drosophila melanogaster* is one such model that has been widely used to study host–microbe interactions (7–10).

Similar to the mammalian intestinal tract, the *Drosophila* gut is equipped with barriers that control bacterial proliferation and prevent microbe-induced damage to the gut epithelia (11, 12). Specifically, ingested pathogens induce two types of effectors that act synergistically to restrict the growth of intestinal microorganisms: AMPs and reactive oxygen species (ROS) (13–15). Infection-induced expression of AMPs in specific regions of the gut is the hallmark of the *Drosophila* immune response (16). The AMP response is regulated by two conserved nuclear factor-kB (NF-κB) pathways: Toll and immune deficiency (Imd) (17, 18). The Imd pathway is initiated in the gut when diaminopimelic (DAP)-type peptidoglycan from bacteria is sensed by the transmembrane recognition receptor PGRP-LC in the ectodermal parts of the gut (foregut and hindgut) or by the intracellular receptor PGRP- LE in the midgut, ultimately leading to the nuclear translocation of the NF-kB transcription factor Relish (19, 20). Activated Relish then induces the expression of immune effectors, like AMPs, that eliminate the pathogens.

With the advent of microbiome research, it became apparent that Imd also responds to commensals and mediates their impact on several physiological processes. Indeed, the relatively simple microbiota of fruit flies, consisting of about 30 phylotypes and dominated by *Lactobacillaceae* and *Acetobacteraceae*, has been shown to have profound effects on intestinal metabolism, immune response, and tissue homeostasis (9, 21, 22). Most of the *Drosophila* microbiota members, similar to pathogens, produce DAP-type PGN—a major elicitor of the Imd pathway. However, the microbiota remains only a mild inducer of the AMP response, suggesting that flies deploy immune tolerance mechanisms to commensals. Indeed, while commensals stimulate a weak AMP response, they induce a strong expression of negative regulators of the Imd pathway (20, 23, 24). These negative regulators, like the PGN-degrading enzymes PGRP-SC1 and PGRP-LB, maintain a low basal level of immunostimulatory PGN, thus preventing the overactivation of the Imd pathway to the gut microbiota (25–27). On the one hand, these negative regulators protect the host from chronic deleterious Imd pathway activation, on the other hand, they prevent a strong AMP response that would target gut commensals. However, during infection, this host–microbiota homeostasis is disrupted as pathogens trigger a transient but strong production of AMPs. These AMPs not only neutralize pathogens but will also target gut commensals. Therefore, the question of how the composition and abundance of intestinal microbiota is affected by infection and how it tolerates the exposure to AMPs remains to be addressed. Similarly, during aging, flies exhibit increased commensal loads despite an elevated AMP response (28, 29), raising the question of how commensals are able to survive in these inflamed environments.

Here, we showed that *Drosophila* microbiota composition and abundance remain stable during infection. Using the dominant *Drosophila* commensal *Lactiplantibacillus plantarum* as a model, we discovered that it does not avoid the immune response but is resistant to cationic AMPs. In a transposon screen, we identified several AMP-sensitive *L. plantarum* mutants with altered cell wall modifications and increased binding of AMPs. The abundance of AMP-sensitive mutants was significantly reduced after infection in wild-type but not in AMP-deficient flies. Taken together, our results demonstrate that the resistance to host AMPs via cell wall modifications is crucial for microbiota resilience during infection.

## Results

### *Drosophila* Microbiota Is Resistant to the Host Intestinal Immune Responses Induced by Oral Infection.

**Fig. 1.**
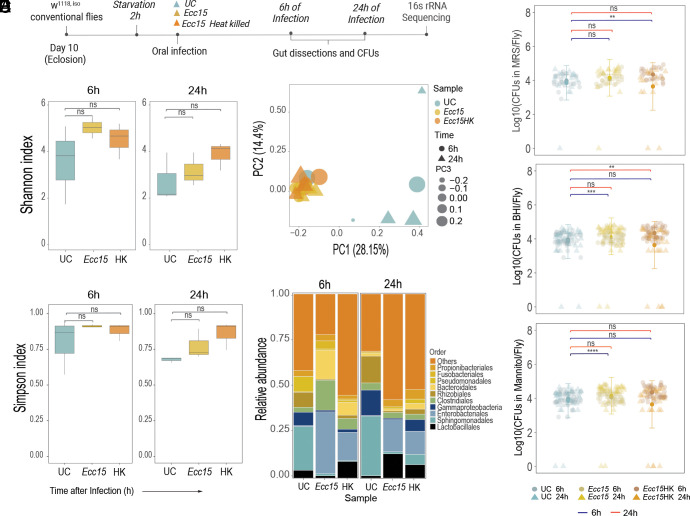
*Drosophila* microbiota is resistant to the host intestinal immune responses induced by oral infection. (*A*) Experimental design to investigate infection’s effect on microbiota composition and abundance. 10-d-old conventional *w^1118^* iso flies were starved for 2h, then either fed sucrose (UC) or infected with alive *Ecc15* (*Ecc15*) or with heat-killed *Ecc15* (*Ecc15*HK). Guts were dissected 6 h and 24 h postinfection for 16S rRNA sequencing or enumeration of bacteria. Unless otherwise stated, n = 3 independent experiments with 20 guts per treatment performed at 6 h and 24 h. (*B* and *C*) Plots showing estimated alpha diversity of the microbial community based on Shannon (*B*) and Simpson (*C*) indexes of the 16S rRNA amplicon sequences (n = 3 replicates). (*D*) Principal coordinate analysis of the microbial community structure (beta diversity) illustrated by unweighted UniFrac distances of 16S rRNA amplicon sequences. (*E*) Order-level relative abundance of 10 dominant OTUs in uninfected (UC) and infected samples. (*F*) Culturable microbiota loads in 10-d-old conventionally reared flies 6 h and 24 h post *Ecc15* live and heat-killed (*Ecc15*HK) oral infections in MRS, BHI, and mannitol agar. Sample size: UC 6 h (n = 26), UC 24 h (n = 18), *Ecc15* 6 h (n = 26), *Ecc15* 24 h (n = 19), *Ecc15*-HK 6 h (n = 25)*, Ecc15*-HK 24h (n = 17). The single dots are mean colony forming unit (CFU) values from pools of n = 5 animals in the Log10 scale. Dot plots and boxplots show median and interquartile range (IQR), and whiskers show either the lower and upper quartiles or range. *P < 0.05, **P < 0.01, ***P < 0.001, ****P < 0.0001. Kruskal–Wallis and Bonferroni post hoc tests were used for statistical analysis.

First, we investigated the impact of intestinal infection on *Drosophila* gut microbiota composition using 16S rRNA sequencing. For this, we orally infected 10-d-old conventional (colonized with native microbiota) wild-type iso (*w^1118 iso^*) flies with alive and heat-killed *Erwinia carotovora (Ecc15)* and dissected guts 6 h and 24 h postinfection for DNA extraction and 16S rRNA sequencing (Fig. 1*A*). Heat-inactivated *Ecc15* induces the IMD pathway but does not directly interfere with commensals. Alpha diversity in 16S rRNA amplicon sequencing indicated that intestinal bacterial community diversity was not significantly different between uninfected controls and flies infected with either live or heat-killed *Ecc15,* as illustrated by Shannon and Simpson indexes (Fig. 1 *B* and *C*) and by additional indexes of alpha diversity (*SI Appendix*, Fig. S1 *A*–*C*). Additionally, we assessed similarities between microbial communities in all samples using beta diversity analyses. Principal coordinate analysis (unweighted UniFrac) showed tight clustering of all infected samples together, indicating similarity of bacterial communities (Fig. 1*D*). A permutation-based, multivariate ANOVA (Adonis) (30, 31) confirmed that all infected samples statistically are not different (pairwise ADONIS: *P*-adjusted_Ecc15 6 h vs. Ecc15 24 h_ = 0.1; *P*-adjusted_Ecc15HK 6 h vs. Ecc15HK 24 h_ = 0.7). In contrast, uninfected samples are more dispersed (Fig. 1*D*), illustrating variability in microbiota composition among individual replicates in uninfected flies. Statistically, however, uninfected samples collected at two timepoints are similar (pairwise ADONIS: *P*-adjusted_UC 6 h vs. UC 24 h_ = 0.6). While uninfected samples cluster separately from infected ones (Fig. 1*D*), statistically they were not different (pairwise ADONIS: *P*-adjusted_UC 6 h vs. Ecc15 6 h_ = 0.1; *P*-adjusted_UC 6 h vs. Ecc15HK 6 h_ = 0.1; *P*-adjusted_UC 24 h vs. Ecc15 24 h_ = 0.3; *P*-adjusted_UC 24 h vs. Ecc15HK 24 h_ = 0.3). Next, we analyzed the relative abundance of the most representative species in our 16S sequencing data. Consistent with previous studies (32, 33), microbial communities in our flies were dominated by the phylum of Firmicutes represented by the families of *Lactobacillaceae* and *Enterococcaceae* and the phylum of Proteobacteria represented by the families of *Sphingomonadaceae* and *Enterobacteriaceae* (*SI Appendix*, Fig. S1*D*). We noticed that infection led to a reduction in *Sphingomonadales* and a stable or even increased abundance of *Clostridiales* and *Lactobacillales* species (Fig. 1*E*). Overall, while there were minor changes in the composition after infection, alpha and beta diversity indexes did not show any statistically significant changes in microbiota composition between uninfected and infected samples.

Next, to corroborate our 16S rRNA sequencing results, we recorded the microbiota loads of whole 10 d conventional flies infected with heat-killed and alive *Ecc15* by plating fly homogenates on media supporting microbiota growth (De Man, Rogosa and Sharpe (MRS), Mannitol, and Brain Heart Infusion (BHI)). Treatment with *Ecc15* did not reduce microbiota load relative to uninfected controls in all tested conditions (Fig. 1*F*). We even observed an increase in microbiota cell numbers on MRS 6 h after heat-killed *Ecc15* infection and on BHI and Mannitol 6 h after live infection (Fig. 1*F*), indicating that total microbiota numbers either stay stable or increase after infection.

**Fig. 2.**
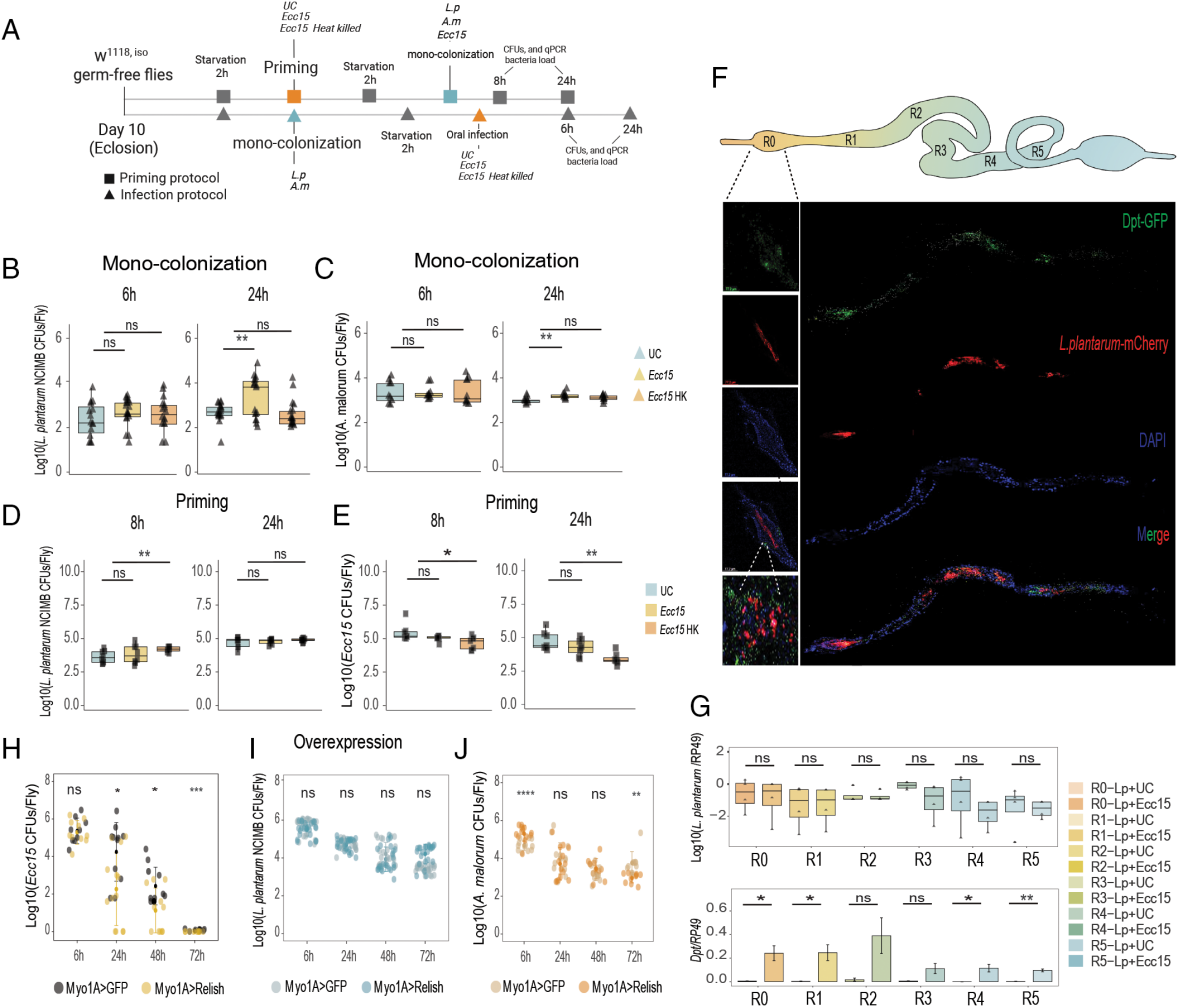
*L. plantarum* is present in the immune-responsive gut regions and is resistant to the IMD pathway effectors. (*A*) Experimental design for monocolonization and priming protocols. Details are specified in Methods section. Monocolonization-Infection: *L. plantarum^NCIMB^* (*B*) and *A. malorum* (*C*) CFUs 6 h and 24 h after the oral infection with *Ecc15*. Priming: *L. plantarum^NCIMB^* (*D*) and *Ecc15* (*E*) loads 8 h and 24 h in flies that were orally primed with *Ecc15* before colonization (n = 8 independent samples per treatment with five flies per sample). The single dots are mean CFU values from pools of n = 5 animals in the Log10 scale. (*F*) Imaging of adult *DptA-GFP Drosophila* gut colonized with *L. plantarum*-mCherry^WJL^ and infected with *Ecc15* (6 h postinfection). The *Left* panel displays a magnification of the proventriculus (R0), a niche for *L. plantarum* (10) and a major immune-responsive gut region. (*G*) *L. plantarum^WJL^* loads recorded by qPCR 6 h after *Ecc15* infection, matched with *DptA* expression per gut region in *w^1118^* iso flies. Bar plots show media and se range (n = 3 independent experiments). (*H*) *Ecc15* loads at 6 h (n1 = 9 and, n2 = 10), 24 h (n1 = 11 and, n2 = 13), 48 h (n1 = 11 and, n2 = 11), and 72 h (n1 = 10 and, n2 = 10) postinfection in *Myo1A-GAL4>UAS-GFP* (n1) and *Myo-GAL4>UAS-Relish* flies (n2). (*I*) CFUs of *L. plantarum^NCIMB^*in *Myo1A-GAL4>UAS-GFP* and *Myo-GAL4>UAS-Relish* flies at 6 h, 24 h, 48 h, and 72 h postcolonization (n = 24 for both genotypes at all time points). (*J*) CFUs of *A. malorum* at 6 h (n1 = 12 and, n2 = 13), 24 h (n1 = 14 and, n2 = 14), 48 h (n1 = 11 and, n2 = 10), and 72 h (n1 = 10 and, n2 = 10) post colonization in *Myo1A-GAL4>UAS-GFP* (n1) and *Myo-GAL4>UAS-Relish* flies (n2). The stats markings in *H*–*J* refer to pairwise comparison between *Myo1A-GAL4>UAS-GFP* and *Myo-GAL4>UAS-Relish.* The single dots are mean CFU values from pools of n = 5 animals in the Log10 scale. Boxplots and dot plots show median and interquartile ranges (IQR); whiskers show either lower or upper quartiles or ranges. *P < 0.05, **P < 0.01, ***P < 0.001, ****P < 0.0001. Kruskal–Wallis and Bonferroni post hoc tests were used for statistical analysis.

In the next set of experiments, we used gnotobiotic flies monocolonized with representative microbiota isolates *L. plantarum* NCIMB or *A. malorum* and measured their persistence during infection with alive and heat-killed *Ecc15* using monocolonization and priming protocols (Fig. 2*A*). In monocolonization protocol, flies were first colonized with microbiota and then infected, while in priming protocol, flies were first infected and then colonized with microbiota. With monocolonization protocol, using colony forming unit (CFU) counts, we found that the loads of *L. plantarum* NCIMB (Fig. 2*B*) and *A. malorum* (Fig. 2*C*) were either stable or even increased 24 h postinfection with alive *Ecc15*. Additionally, we used qPCR-based quantification with bacteria-specific primers and confirmed stable persistence of *L. plantarum* NCIMB (*SI Appendix*, Fig. S2*A*) and increased abundance of *A. malorum* (*SI Appendix*, Fig. S2*B*) during infection. This was also true for the different *L. plantarum* strain (NC8) that we tested [*SI Appendix*, Fig. S2*C* (CFUs), S2D (qPCR)].

Next, we investigated whether intestinal immune activation prior to colonization (priming) will affect the ability of representative commensals to colonize the gut. To address the effect of immune priming, we infected wild-type germ-free flies with either alive or heat-killed *Ecc15* and performed colonization with certain microbiota members (Fig. 2*A*). *L. plantarum* NCIMB showed an increase in CFUs after the *Ecc15* heat-killed treatment at 8 h and no significant changes under the other conditions (Fig. 2*D*). We confirmed these results by qPCR (*SI Appendix*, Fig. S2*E*) and for different *L. plantarum* strain (NC8) [*SI Appendix*, Fig. S2*F* (CFUs), S2G (qPCR)]. Importantly, when we infected *Ecc15*- primed flies with *Ecc15*, we observed reduced *Ecc15* loads compared to control flies using both CFU-based (Fig. 2*E*) and qPCR-based measurements (*SI Appendix*, Fig. S2*H*). *P. entomophila (Pe)* behaved in a similar way (*SI Appendix*, Fig. S2*I*). These results prove that priming 1) induces an immune response and 2) that this immune response is effective against pathogens but not the microbiota members that we tested.

Altogether, our results demonstrate that infection causes only minor changes in the composition of the *Drosophila* microbiota and that the dominant gut commensals can persist in the gut during active immune responses induced by pathogens.

### *L. plantarum* Is Present in the Immune-Responsive Gut Regions.

Next, we focused on one of the dominant commensals in our flies, *L. plantarum*, to understand how it persists in the gut during infection. One hypothesis we had is that *L. plantarum* might simply avoid regions where the immune system is active and thereby survive the immune challenge. This scenario is possible, considering that the *Drosophila* gut is regionalized (Fig. 2*F*) and that the gut regions differ in the intensity of the immune response (34). We took several approaches to investigate this avoidance hypothesis. First, we infected flies that had been monocolonized with *L. plantarum* with *Ecc15* and dissected guts 6 h postinfection. The dissected guts were separated into six regions. We then performed qPCR to quantify *L. plantarum* and measure activation of the Imd pathway in the same intestinal region. As shown in Fig. 2*G*, *Ecc15* infection potently induced *Diptericin A* expression (Imd pathway readout) in all gut regions, except R2 and R3. *L. plantarum* was detected in all the gut regions and its abundance was not affected by infection. Consistently, we also did not find increased *L. plantarum* loads in the gut regions (R2 and R3) lacking *DptA* induction after infection. Overall, this suggests that *L. plantarum* colonizes all *Drosophila* gut regions, regardless of the level of their active immune response. Additionally, we used a microscopy approach to visualize both immune activation and *L. plantarum* localization in the gut. Specifically, we colonized *DptA-GFP* reporter flies with *L. plantarum-mCherry* bacteria and visualized the localization of both 6 h after *Ecc15* infection. We observed (Fig. 2*F*) an abundant localization of *L. plantarum-mCherry* in the foregut region where *Dpt-GFP* expression was also very pronounced. A similar colocalization pattern was also seen in the other gut regions, confirming that *L. plantarum* does not avoid regions with active immune responses. Finally, we genetically overactivated the Imd pathway in enterocytes, a major intestinal cell type, by overexpressing the transcription factor *Relish* and assessed the persistence of *L. plantarum, A. malorum,* and *Ecc15* in these flies*. Relish* overexpression potently induced Imd pathway activation to a similar level as *Ecc15* infection as demonstrated by the expression of three Imd pathway target genes (*SI Appendix*, Fig. S3*A*). While such intestine-wide immune activation significantly reduced the colonization of the gut by *Ecc15* in both CFU-based (Fig. 2*H*) and qPCR-based assays (*SI Appendix*, Fig. S3*B*), it did not significantly affect the abundance of *L. plantarum* [Fig. 2*I* (CFUs) and *SI Appendix*, Fig. S3*C* (qPCR)] across several timepoints. We used an additional method to genetically activate immune response, namely *Imd* overexpression in the gut, and observed that *L. plantarum* numbers remained stable also in these flies (*SI Appendix*, Fig. S3*D*). Behavior of another gut commensal, *A. malorum,* was time-dependent with abundance being either not affected by *Relish* overexpression (24 h, 48 h postcolonization), significantly reduced (72 h post-colonization), or significantly increased (6 h postcolonization) by genetic immune activation (Fig. 2*J*). Overall our results suggest that gut commensals have a mechanism other than avoidance to withstand the action of intestinal immune defenses.

### *Drosophila* Commensals Are Resistant to Cationic AMPs In Vitro.

**Fig. 3.**
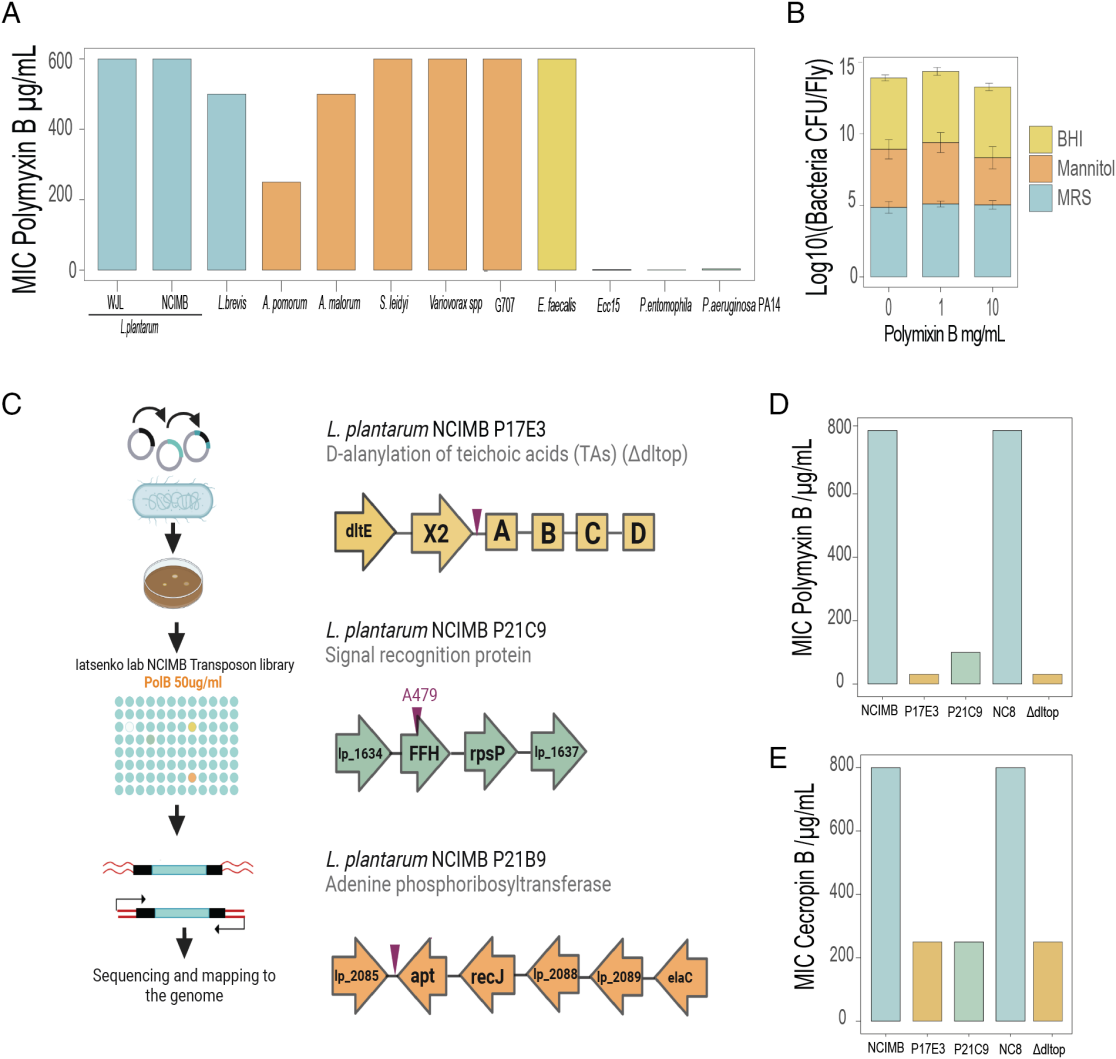
*Drosophila* microbiota is resistant to the AMPs in vitro. (*A*) Sensitivity of *Drosophila* microbiota members and pathogens to Polymyxin B in Minimal Inhibitory Concentration (MIC) assay. (*B*) Amount of culturable microbes derived from 10-d-old flies able to grow on MRS, Mannitol, and BHI agar plates supplemented with Polymyxin B. (*C*) Scheme of the transposon screening in *L. plantarum^NCIMB^*for polymyxin B-sensitive mutants and location of transposon insertions. Genomic organization of *dlt* operon in *L. plantarum^NCIMB^*, insertion between the *dltX* and *dltA* genes; lp_1634 operon, where the insertion hits at the end of *ffh* gene, and *lp_2085* where the insertion hits in between the *lp_2085* operon and *apt*. (*D* and *E*) MIC of *L. plantarum^NCIMB^,* P17E3, P21C9 transposon mutants, and *L. plantarum^NC8^* and ∆*dltop* mutant in broth supplemented with either Polymyxin B (*D*) or Cecropin B (*E*). Bar plots show mean values. MIC experiments were repeated three times with identical results; therefore, no error bars are shown.

We hypothesized that the *Drosophila* microbiota members are resistant to host AMPs and, therefore, can survive infection-induced immune responses. Since it is not possible to obtain in vitro the exact combination of AMPs that is produced by intestinal cells of the fruit fly, we decided to test the readily available cationic antimicrobial peptide polymyxin B. It has been widely used to model AMP sensitivity and mimics the action of some *Drosophila* AMPs (5, 35, 36). Minimal inhibitory (MIC) test showed that typical *Drosophila* gut commensals like *L. plantarum*, *L. brevis*, *A. malorum,* and *E. faecalis* are resistant to polymyxin B and still grow at the highest tested concentrations. In contrast, oral pathogens of fruit flies, including *Ecc15*, *P. entomophila,* and *P. aeruginosa* were sensitive and did not grow even at the lowest concentration of polymyxin B (Fig. 3*A*). Additionally, to assess how common resistance to AMPs is among *Drosophila* microbiota members, we estimated the proportion of polymyxin resistant microbes in our lab flies by plating fly homogenates on growth media supplemented or not with the antibiotic. While polymyxin supplementation had no effect on the number of bacteria growing on MRS and BHI, we observed a slight reduction in the number of mannitol-growing bacteria in the presence of the antibiotic. This indicates that the majority of culturable microbes in our flies are resistant to polymyxin B (Fig. 3*B*) and thus likely also to some of the *Drosophila* AMPs.

### Identification of AMP-Sensitive *L. plantarum* Mutants.

Next, we investigated the genetic bases of resistance to AMPs in *L. plantarum,* which is the dominant microbe in our lab flies and not only persisted but also expanded after infection. We took a classical unbiased forward genetic approach in which a library of bacterial mutants was screened for sensitivity to polymyxin B. First, we generated a random transposon mutant library in *L. plantarum* NCIMB8826 strain, which has a high transformation efficiency in contrast to *Drosophila* isolates. We mutagenized *L. plantarum* NCIMB8826 using the previously described P_junc_-TpaseIS *_1223_* transposon mutagenesis system (37, 38) and randomly selected and stocked 3000 colonies as individual clones at −80 °C. We screened this library for mutants that were unable to grow in the presence of 50 µg/mL of polymyxin B, which is at least 10 times lower than the wild-type *L. plantarum* could survive (Fig. 3*C*). Under these selective conditions, initially we identified 15 potential candidates. However, we could not confirm the sensitivity for six of them in the second test, and another six candidates exhibited impaired growth even in the absence of polymyxin B. Therefore, only three mutants passed the selection criteria and were used for the identification of transposon insertion site. In one of these mutants (P21B9), the transposon insertion happened in an intergenic region between the ORFs encoding adenine phosphoribosyltransferase (*apt*) and a transcription regulator (*lp_2085*) (Fig. 3*C*). Given that such an intergenic insertion can affect either an upstream or downstream ORF, or both, we decided not to consider this complex case in this study. In mutant P21C9, the transposon hit the *ffh* (fifty-four homologue) gene which is part of the signal recognition particle pathway (SRP) necessary for protein translocation, secretion, and membrane incorporation (39). In the P17E3 mutant, the transposon was inserted between the *dltX and dltA* genes of the *dlt* operon (Fig. 3*C*). The *dlt* operon is responsible for the esterification of wall teichoic acids (WTAs) with d-alanine, thus reducing their negative charge and attraction of cationic AMPs to the bacterial cell wall (40, 41). Consequently, mutants of the *dlt* operon are susceptible to AMPs, which we confirmed here for *L. plantarum*. Using a MIC test, we verified that P17E3 and P21C9 are indeed several times more sensitive to polymyxin B than wild-type bacteria (Fig. 3*D*). Interestingly, our P17E3 transposon mutant exhibited the same level of sensitivity as a previously generated *L. plantarum* deletion mutant lacking the entire *dlt* operon (*∆dltop)* (Fig. 3 *D* and *E*). P17E3, *L. plantarum ∆dltop*, and P21C9 mutants were also more susceptible to the insect AMP cecropin B (Fig. 3*E*), confirming that our *L. plantarum* mutants are sensitive to other cationic AMPs and not specifically to polymyxin B. We decided to further characterize P21C9 (*ffh*) and P17E3 (*dltop*) mutants and investigate the reason for their sensitivity to AMPs and its consequences for the interactions with the host.

### Disruption of the *dlt* Operon and the *ffh* Gene Increases Binding of Cationic AMPs to the Cell Surface.

**Fig. 4.**
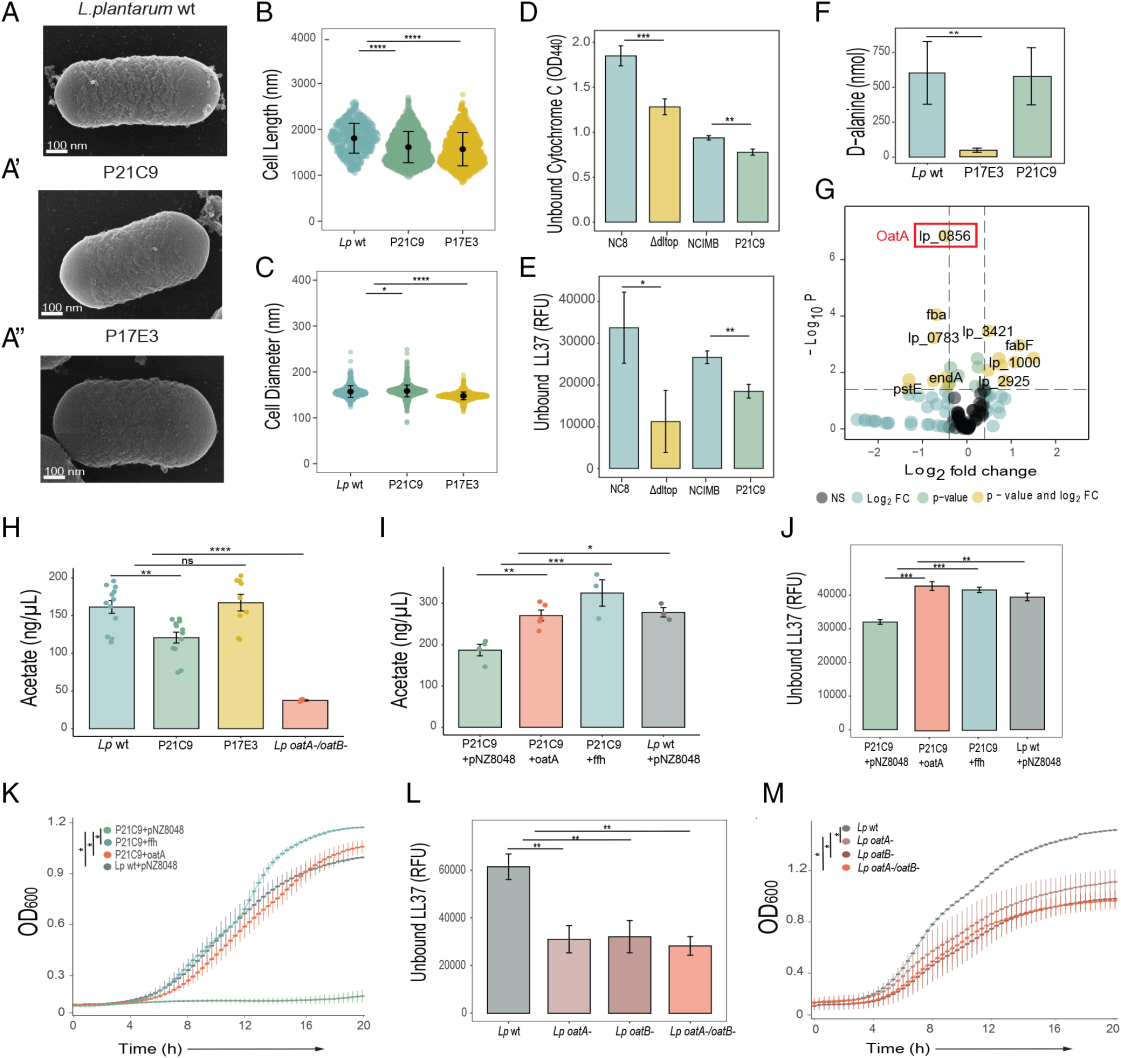
Microbiota resistance to the AMPs is driven by O-acetylation of peptidoglycan and D-alanylation of the teichoic acid. (*A*) Scanning electron microscopy image of *L. plantarum^NCIMB^*, P21C9 (A’), and P17E3 (A”) cell. (*B*) Cell length and (*C*) cell diameter of *L. plantarum^NCIMB^* (n = 612), P21C9 (n = 1,535), and P17E3 (n = 1,006). Individual dots show single cell record. Violin dot plots show median and interquartile ranges. (*D* and *E*) Binding of *L. plantarum^NCIMB^*, P21C9, and P17E3 cells to Cytochrome C (*D*) and to fluorescently labeled antimicrobial peptide LL37 (*E*) (n = 3 independent experiments). Quantity of remaining cytochrome C (quantified by measuring OD440) or fluorescently labeled antimicrobial peptide LL37 (quantified by measuring fluorescence and expressed as Relative Fluorescent Units, RFU) in the solution after incubation with indicated bacteria. (*F*) HPLC quantification of D-alanine released by whole cells of *L. plantarum^NCIMB^*, P21C9, and P17E3 (n = 6 independent cultures). Bar plots show mean and SEM. (*G*) Differential analysis of secreted and membrane-bound proteins in *L. plantarum^NCIMB^* and P21C9. Lines in volcano plot show the p value cut-off (0.05) and fold change cut-off (0.5) (n = 3 independent samples). The most significantly underrepresented protein (OatA) in the supernatant of P21C9 mutant is highlighted in red. (*H*) Quantification of acetate released from peptidoglycan extracted from *L. plantarum^NCIMB^* (n = 12), P21C9 (n = 12), P17E3 (n = 9), and *L. plantarum^oatA-/oatB-^* (n = 3). (*I*) Quantification of acetate released from peptidoglycan extracted from P21C9 mutant overexpressing *oatA* (n = 5) or *ffh* (n = 3). P21C9 mutant (n = 4) and wild-type *L. plantarum* (n = 3) containing empty pNZ8048 plasmid were used as controls. Bar plots show mean and SEM. (*J*) Binding of the indicated strains to labeled antimicrobial peptide LL37 (n = 3 independent experiments). (*K*) Kinetics of the growth of P21C9+pNZ8048 (empty plasmid), P21C9+*ffh*, P21C9+*oatA*, and *L. plantarum^NCIMB^*+ pNZ8048 in MRS media supplemented with Polymyxin B (n = 3 independent experiments). Mean and SEM are shown. (*L*) Binding of *L. plantarum^NCIMB^L. plantarum^oatA-^*, *L. plantarum^oatB−^*, and *L. plantarum^oatA−/oatB−^* to labeled antimicrobial peptide LL37. Bar plots show mean and SEM (n = 3 independent experiments). (*M*) Kinetics of the growth of *L. plantarum^NCIMB^L. plantarum^oatA−^*, *L. plantarum^oatB−^*, and *L. plantarum^oatA−/oatB−^*in MRS media supplemented with Polymyxin B, (n = 3 independent experiments). Mean and SEM are shown. *P < 0.05, **P < 0.01, ***P < 0.001, ****P < 0.0001. Kruskal–Wallis and Bonferroni post hoc tests were used for statistical analysis. Simple linear regression analysis was performed for the kinetics analysis.

Considering that the cell surface is a key mediator of AMP–bacteria interactions, we used scanning electron microscopy to explore cell morphology and measure cell parameters of the P17E3 and P21C9 mutants. We did not observe any obvious morphological alternations in both mutants (Fig. 4*A*), with the exception that their cells appeared smaller compared to wild-type cells. Quantification of cell length confirmed that P17E3 and P21C9 cells are significantly shorter than wild-type cells (Fig. 4*B*). Consistent with a previous study (42), we detected a significant reduction in the width of P17E3 cells (Fig. 4*C*), while P21C9 cells were slightly wider compared to wild-type cells (Fig. 4*C*). Next, we investigated whether the observed morphological alternations affected the cell surface properties of the P21C9 and P17E3 mutants. Specifically, we measured cell surface charge which is a critical parameter in bacterial sensitivity to AMPs. Surface charge was estimated indirectly by quantifying the amount of cationic molecules that remain in the solution after incubation with bacteria. Both P21C9 and *∆dltop* mutants had significantly less unbound cationic cytochrome C in the solution compared to wild-type bacteria, indicating an increased binding and negative surface charge (Fig. 4*D*). Using the same principle, we found that binding of the fluorescently labeled cationic AMP 5-FAM-LC-LL37 was increased to P21C9 and *∆dltop* cells (Fig. 4*E*). Next, we decided to quantify D-alanine esterification of WTAs, due to its major role in regulating surface charge. Using HPLC, we confirmed that P17E3 cells, consistent with the function of the *dlt* operon, have reduced levels of D-alanine esterification of WTAs (Fig. 4*F*). In the P21C9 mutant, we detected an amount of D-ala released from WTAs that was comparable to wild-type bacteria (Fig. 4*F*), suggesting that a different cell wall modification affects the surface charge in this mutant. Considering that the disrupted *ffh* gene in the P21C9 mutant is part of the SRP necessary for protein translocation and secretion, we hypothesized that the P21C9 mutant might be impaired in the secretion of certain proteins necessary for cell wall biogenesis or modifications as previously reported in different bacteria (43–45). To test this hypothesis, we performed a proteomic analysis of secreted and surface-associated proteins in the P21C9 mutant and wild-type bacteria. In total, we detected 111 proteins; however, only 25 proteins passed p value and fold change significance cutoffs (Dataset S1). Majority of proteins (21 out of 25) showed a reduced abundance in the P21C9 mutant, which would be consistent with the theory of impaired protein secretion. Among proteins with reduced abundance, two acyltransferases [OatA (lp_0856) and OatB (lp_0925)] implicated in PGN O-acetylation (46) caught our attention (Fig. 4*G*). Since PGN O-acetylation mediates sensitivity to lysozyme (46, 47), we hypothesized that the P21C9 mutant might be sensitive to AMPs due to reduced PGN O-acetylation as a consequence of reduced secretion of acyltransferases. We extracted PGN from mutant and wild-type bacteria and quantified the amount of acetate associated with PGN. Consistent with our hypothesis, we detected significantly reduced PGN acetylation levels in the P21C9 mutant (Fig. 4*H*) and no significant change in P17E3. An *L. plantarum* mutant lacking both acyltransferases (46) showed the expected lack of PGN acetylation, validating our assay (Fig. 4*H*). Importantly, we could rescue PGN acetylation in the P21C9 mutant to wild-type level by *ffh* or *oatA* overexpression (Fig. 4*I*). *OatA* overexpression also reduced the binding of cationic AMPs to P21C9 cells (Fig. 4*J*) and rescued P21C9 mutant sensitivity to polymyxin B (Fig. 4*K*). These results suggest that disruption of *ffh* leads to a decreased secretion of the OatA protein, consequently reducing PGN acetylation, increasing negative surface charge and sensitivity to AMPs in the P21C9 mutant. Consistent with this, *L. plantarum* mutants lacking acyltransferases showed increased binding to 5-FAM-LC-LL37 (Fig. 4*L*) and enhanced susceptibility to polymyxin B (Fig. 4*M*). Together, these results establish O-acetylation as an additional PGN modification mediating bacterial sensitivity to cationic AMPs via surface charge alternations.

### Resistance to AMPs Is Essential for *L. plantarum* Persistence in the Gut during Immune Activation.

**Fig. 5.**
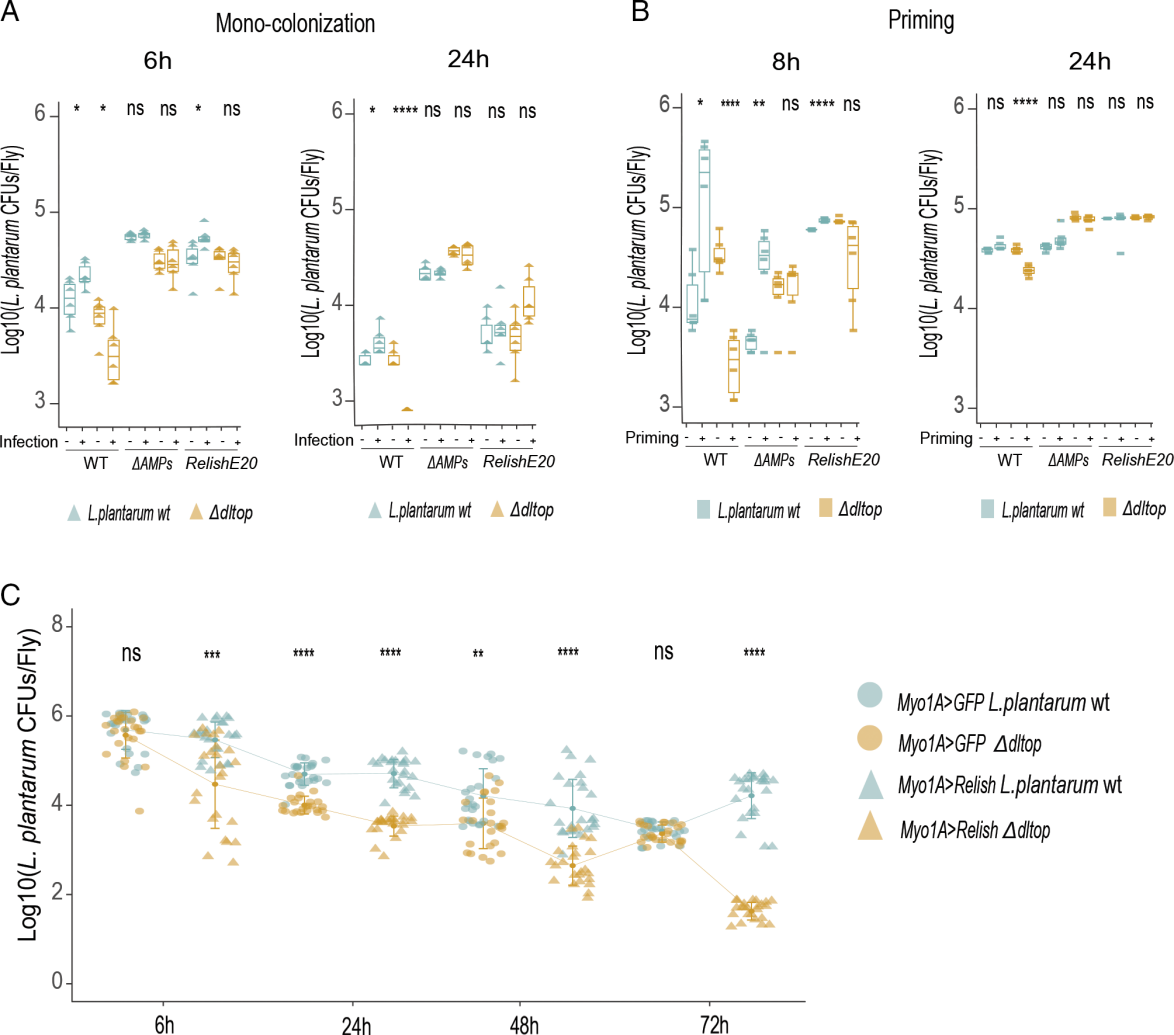
*L. plantarum delta* operon genes are essential to colonize and persist in the gut during infection. (*A*) *L. plantarum^NC8^* and *L. plantarum^∆dltop^* loads in wild-type, ∆*AMPs,* and *Relish^E20^* flies 6 h and 24 h after infection with *Ecc15* (n = 6 independent samples per treatment with 5 flies per sample). (*B*) *L. plantarum^NC8^* and *L. plantarum^∆dltop^* loads after the priming with *Ecc15* at 8 h and 24 h in wild type, ∆*AMPs,* and *Relish^E20^* (n = 6 independent samples per treatment with five flies per sample). (*C*) *L. plantarum^NC8^* and *L. plantarum^∆dltop^* loads in *Myo1A-GAL4>UAS-GFP* and *Myo-GAL4>UAS-Relish* flies at 6 h, 24 h, 48 h, and 72 h after colonization (n = 24 samples per treatment with five flies per sample). Individual tringles, squares, and dots show mean CFU values from pools of n = 5 animals in the Log10 scale. Boxplots and dot plots show median and interquartile ranges (IQR); whiskers show either lower or upper quartiles or ranges. *P < 0.05, **P < 0.01, ***P < 0.001, ****P < 0.0001. Kruskal–Wallis and Bonferroni post hoc tests were used for statistical analysis.

Next, we investigated the persistence of AMP-sensitive *L. plantarum* mutants in the *Drosophila* gut. First, we used gnotobiotic flies monocolonized with wild-type *L. plantarum* and *∆dltop* mutant and measured their abundance in uninfected and *Ecc15*-infected flies (Fig. 5*A*). The load of the *∆dltop* mutant was significantly lower compared to wild-type *L. plantarum* 6 h and 24 h post *Ecc15* infection. However, the *∆dltop* mutant colonized uninfected flies as efficiently as wild-type *L. plantarum*, suggesting that the decline after infection is not due to a general incapacity to persist in the gut, but rather due to sensitivity to immune activation. Supporting this, the load of the *∆dltop* mutant did not decline after infection in flies lacking AMPs (∆*AMP)* or *Relish* (Fig. 5*A*). Second, using a priming approach (Fig. 5*B*), we observed a similar result: reduced abundance of *∆dltop* mutant in infected wild-type flies and rescue of this phenotype in ∆*AMP* and *Relish* mutant flies. Finally, genetic overactivation of the Imd pathway in the gut resulted in a significant decrease in *∆dltop* mutant levels compared to control flies and wild-type *L. plantarum* at all time points tested (Fig. 5*C*). At two timepoints (24 h and 48 h), the numbers of *∆dltop* mutant were lower compared to wild-type *L. plantarum* also in control flies overexpressing GFP. However, the difference between *∆dltop* and wild-type *L. plantarum* at the same two timepoints became even stronger in flies overexpressing *Relish* suggesting that sensitivity to AMPs at least partly contributes to the reduced abundance of *∆dltop* mutant. The P21C9 (*ffh*) mutant behaved similar to *∆dltop*—it stably colonized uninfected flies but failed to persist in the gut after *Ecc15* infection (*SI Appendix*, Fig. S4*A*), after priming at 24h timepoint (*SI Appendix*, Fig. S4*B*) or genetic activation of the immune response (*SI Appendix*, Fig. S4*C*). Persistence of the P21C9 mutant during infection was restored in ∆*AMP* and *Relish* flies (*SI Appendix*, Fig. S4 *A* and *B*), proving that AMPs at least in part are responsible for the clearance of AMP-sensitive mutants during infection. Overall, our results indicate that resistance to host AMPs is essential for *L. plantarum* to stably persist in the inflamed gut environment.

### Resistance to AMPs Is Essential for *L. plantarum* Persistence in the Gut during Aging.

**Fig. 6.**
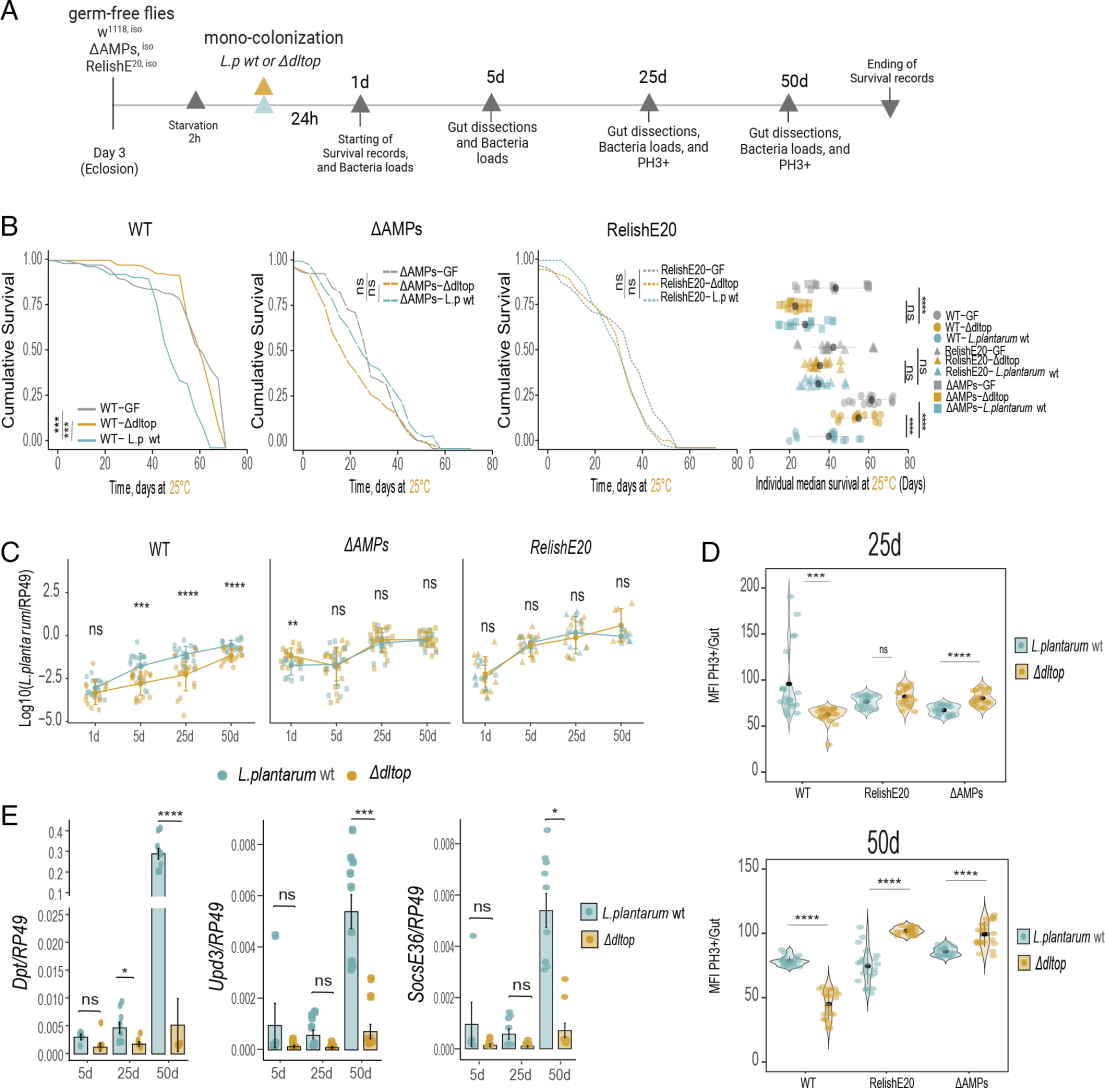
*L. plantarum* resistance to the AMPs is a crucial regulator of the onset of *Drosophila* aging. (*A*) Experimental design of aging experiment. Germ-free, 3-d-old *w^1118^* iso, ∆*AMPs* and *Relish^E20^* flies were starved for 2 h and then monocolonized by feeding with *L. plantarum^NC8^* or *L. plantarum^∆dltop^*(OD50) for 24 h. Flies were flipped to fresh vials every 2 d and sampled at indicated timepoints. (*B*) On the *Left* panel Kaplan–Meier survival curves of monocolonized flies with either *L. plantarum^NC8^* or *L. plantarum^∆dltop^*, germ-free flies were used as control. *P* values were obtained from stratified groups of fly genotypes, the log-rank test was applied to the survival curves per stratified group (n = 3). *Right* panel shows Median Survival in monocolonized flies. Single dots show the median survival per sample, germ-free flies were used as control. (*C*) *L. plantarum^NC8^* (n1) or *L. plantarum^∆dltop^* (n2) loads in wild-type 1-d- (n1 = 15, n2 = 16), 5-d- (n1 = 20, n2 = 19), 25-d- (n1 = 19, n2 = 19), 50-d-old (n1 = 16, n2 = 16); in *∆AMPs* 1-d- (n1 = 14, n2 = 14), 5-d- (n1 = 20, n2 = 19), 25-d- (n1 = 18, n2 = 20), 50-d-old (n1 = 14, n2 = 18), and in *Relish^E20^* 1-d- (n1 = 13, n2 = 13), 5-d- (n1 = 11, n2 = 14), 25-d- (n1 = 12, n2 = 11), 50-d-old (n1 = 9, n2 = 7) monocolonized flies. Individual dots show mean load values determined by qPCR from pools of n = 5 animals in the Log10 scale. (*D*) Intestinal stem cell proliferation, indicated by median fluorescence intensity (MFI) of Alexa fluor 555 positive phospho-histone H3-positive cells per gut in wild-type, *∆AMPs,* and *Relish^E20^* flies monocolonized with *L. plantarum^NC8^* or *L. plantarum^∆dltop^* at 25 d or 50 d (n = 21 guts per sample). (*E*) Gene expression of *DptAUpd3Socs36E* in 5-d- (n1 = 5, n2 = 7), 25-d- (n1 = 9, n2 = 7), and 50-d- (n1 = 10, n2 = 10) wild-type flies monocolonized with *L. plantarum^NC8^* or *L. plantarum^∆dltop^*. Individual dots show gene expression per 20 guts of female flies. Bar plots show mean and SEM. Violin plots show median and interquartile ranges (IQR). *P < 0.05, **P < 0.01, ***P < 0.001, ****P < 0.0001. Kruskal–Wallis and Bonferroni post hoc tests were used for statistical analysis.

Finally, we investigated what happens with long-term persistence of AMP-sensitive mutants and how they impact aging phenotypes. The main motivation to investigate such age-related phenotypes is that the microbiota load is known to increase with age despite increased expression of AMPs. We hypothesized that microbiota resistance to host AMPs is essential to survive this age-associated immune activation. To test this hypothesis, we generated gnotobiotic wild-type, *Relish*, ∆*AMP* flies monocolonized with wild-type *L. plantarum* and *∆dltop* bacteria by feeding flies with a single dose of bacteria at the beginning of the experiment and scored the lifespan, bacterial load, and several aging hallmarks (Fig. 6*A*). Wild-type flies colonized with the *∆dltop* mutant lived longer compared to flies colonized with wild-type bacteria (Fig. 6*B*). Both ∆*AMP* and *Relish* mutants were short-lived compared to wild-type flies and there was no significant difference in the lifespan between wild-type and *∆dltop* mutant-colonized treatments (Fig. 6*B*). As expected, the bacterial load increased with age in all tested treatments (Fig. 6*C*). However, while the *∆dltop* mutant reached significantly lower density in the guts of wild-type flies, it colonized guts of ∆*AMP* and *Relish* mutants as efficiently as the wild-type strain (Fig. 6*C*), suggesting that AMPs reduce *∆dltop* load in wild-type flies. Consistent with the observed reduced lifespan and elevated bacterial load, ∆*AMP* and *Relish* mutants exhibited excessive intestinal dysplasia as illustrated by stem cell overproliferation (measured by PH3 staining) particularly after colonization with the *∆dltop* mutant (Fig. 6*D*). In contrast, in wild-type flies, intestinal dysplasia was significantly reduced in *∆dltop-*colonized flies when compared to flies colonized with wild-type *L. plantarum*. Consistent with increased stem cell proliferation, the JAK-STAT pathway, as one of the major drivers of stem cell differentiation, showed increased activity with aging in the guts of flies colonized with wild-type *L. plantarum* compared to *∆dltop-* colonized flies (Fig. 6*E*, *upd3* and *Socs36E* expression). Similar results were also observed for the activation of the Imd pathway (Fig. 6*E*, *Dpt* expression). These results suggest that AMPs are responsible for controlling commensal load during aging, consistent with the findings of Hanson and Lemaitre (48), and that resistance to AMPs is essential for commensals persistence in the inflamed gut environment of aging flies.

## Discussion

In this study, we show that infection—one of the frequent perturbations occurring in the digestive tract, had little impact on *Drosophila* microbiota composition and abundance. We identified resistance to AMPs as a key feature of microbiota resilience during intestinal inflammation. Thus, our work reveals that in addition to host immune tolerance to the microbiota, commensal-encoded resilience mechanisms are necessary to maintain a stable host–microbiota association during inflammation.

The intestinal immune response is regionalized in *Drosophila* and other insects (34). This regionalization leads to the formation of gut regions with strong expression of negative regulators of the immune response, thus creating a protective zone for symbiotic microbes (20, 49). Moreover, many insects are equipped with specialized symbiotic organs—bacteriomes, where symbionts are maintained. Bacteriomes allow the host to create a favorable environment for symbionts but also to keep them under control and protect them from perturbations, like infections (50). While compartmentalization is an efficient strategy to protect the symbionts from the immune response, we could detect *L. plantarum* in gut regions with strong AMP expression, suggesting that *L. plantarum* does not hide from the effectors in protective gut zones. Instead, our results support a hypothesis that resistance to AMPs mediates commensal resilience during gut inflammation. Using a genetic screen in *L. plantarum*, we identified several determinants of AMP resistance. One of the identified AMP-sensitive mutants had a transposon insertion in the *dlt* operon, which has been previously implicated in the sensitivity to AMPs in several Gram-positive bacteria (40). Consequently, *dlt* mutants in several pathogens exhibit attenuated virulence (24, 51, 52). The *dlt* operon was also found to be essential for the commensal establishment in the host gut, namely for *L. reuteri* in the mouse intestine (53) and for *L. casei* in the rabbit gut (54). Attieh et al reported that the *L. plantarum dlt* mutant is impaired in the colonization of the *Drosophila* gut and that it triggers a stronger immune response compared to wild-type *L. plantarum* (24). Our results similarly support the essential role of the *dlt* operon in *L. plantarum* in colonizing the *Drosophila* gut. However, it was especially important during an immune challenge because the *dlt* operon mutant colonized uninfected flies as efficiently as wild-type *L. plantarum*. A functional *dlt* operon is also required for *L. plantarum* to promote larval growth of *Drosophila* under chronic undernutrition (42), illustrating multiple essential roles of WTA D-alanylation in host–microbe interactions. It is intriguing that the same cell wall modification is crucial for the stable persistence of bacteria in the gut, immunomodulation, and to confer a beneficial impact on the host. This pleiotropy of WTA D-alanylation results in the scenario where commensals lacking D-alanylated WTAs are better sensed by PGRPs and trigger a strong immune response which will eventually eliminate such commensals due to their sensitivity to AMPs. Commensals with D-alanylated WTAs, however, will promote host tolerance mechanisms and will establish a stable association with the host. Thus, D-alanylated WTAs might be used as a signal to recognize beneficial commensals and trigger either tolerance or immune response.

In addition to the *dlt* operon, we identified another mediator of bacterial sensitivity to AMPs—*ffh,* which is an integral part of the SPR. Apart from being important for virulence (55), the role of the SPR pathway in microbial interactions with the host has not been studied. Such a role is very likely, considering that SPR pathway mutants in different bacteria are impaired in the secretion and translocation of proteins necessary for adhesion, biofilm formation, PGN, and cell wall biosynthesis (43–45). Disruption of the SPR pathway likely has a pleiotropic effect on bacterial physiology and could alter sensitivity to AMPs in several ways. Our results, however, support a prominent role of reduced PGN O-acetylation due to reduced secretion of acyltransferases in the sensitivity of the P21C9 (*ffh*) mutant to AMPs. While PGN O-acetylation is a well-established mechanism of bacterial resistance to lysozyme (47), we found that it also mediates sensitivity to cationic AMPs likely by altering the surface charge. It would be intriguing to explore the extent to which the role of PGN O-acetylation in AMP sensitivity is conserved among bacteria.

Earlier studies showed an essential role of AMPs in shaping *Drosophila* gut communities. For instance, Marra et al found that the abundance of multiple commensals, particularly *Acetobacter sp*, is increased in ∆*AMP* mutant flies, supporting a prominent role of AMPs in controlling *Acetobacter* species (29), which, as we showed here, are more sensitive to AMPs than other fly commensals. Despite the increased sensitivity, *A. malorum* still stably colonized flies during infection, suggesting that commensals rely on additional mechanisms besides resistance to AMPs to survive in an inflamed host environment. These mechanisms, which could include priming by AMPs (56), remain to be investigated. Previously, the role of AMPs in controlling the abundance of *L. plantarum* in the fly gut was found to be less pronounced and was evident only in *L. plantarum* monoassociated flies but not when additional community members were present (29). We also observed elevated *L. plantarum* abundance in some cases in ∆*AMP* mutants, suggesting that while *L. plantarum* is resistant to some cationic AMPs like polymyxin B and CecB, it is very likely that there are AMPs or their combinations to which *L. plantarum* is sensitive. Overall, our results support the current view that AMPs control gut commensals. However, some of the microbiota members are more resistant to AMPs, allowing these microbes to better colonize the host during infection. In turn, the host likely relies on additional means to control these commensals and maintain a balanced microbiome. Lysozymes and ROS are among the likely suspects that would be interesting to investigate in the future (57).

Collectively, our work shows that AMP resistance via cell wall modifications historically associated with pathogen virulence is also essential to maintain stable microbiota–host interactions. This adds more evidence that host–symbiont and host–pathogen associations are mediated by the same molecular dialog (58). Further elucidation of the mechanisms of microbiota resilience during inflammation and generality of such mechanisms will be an exciting future endeavor.

## Materials and Methods

### *Drosophila* Stocks and Rearing.

The following *Drosophila* stocks used in this study were kindly provided by Dr. Bruno Lemaitre: DrosDel *w^1118^* iso; *Relish^E20^* iso; *∆AMP* iso; *UAS-Relish*; *UAS-CecA*; *w;Myo1A-Gal4, tub-Gal80TS, UAS-GFP*; *w;esg-Gal4, tub-Gal80TS, UAS-GFP* (14, 23, 48). The following stocks were obtained from the Bloomington Drosophila Stock Center: w^*^; P{DptA-GFP.JM863}D3-2 P{DptA-GFP.JM863}3-4 (55709); *UAS-mCD8::GFP* (32185). The stocks were routinely maintained at 25 °C with 12/12 h dark/light cycles on a standard cornmeal-agar medium: 3.72 g agar, 35.28 g cornmeal, 35.28 g inactivated dried yeast, 16 mL of a 10% solution of methylparaben in 85% ethanol, 36 mL fruit juice, and 2.9 mL 99% propionic acid for 600 mL. Food for germ-free flies was supplemented with ampicillin (50 µg/mL), kanamycin (50 µg /mL), tetracyclin (10 µg/mL), and erythromycin (10 µg /mL). Fresh food was prepared weekly to avoid desiccation. Female flies were used for all experiments. Bacterial strains used in this study are listed in *SI Appendix*, Table S1.

### Generation and Screening of Random Transposon Mutant Library in *L. plantarum* NCIMB8826.

*L. plantarum* transposon mutagenesis was performed using the P_junc_-TpaseIS*_1223_* system as previously described (37, 38). Protocols from the same references were used for mapping of transposon insertion sites. Detailed procedures are explained in *SI Appendix*. Primers used in this study are listed in *SI Appendix*, Table S2.

### Regionalization of *Drosophila* Gut.

*w^1118^* iso germ-free flies were monocolonized with *L. plantarum* for 24 h and infected with *Ecc15* for 6 h. Eighty guts per sample were dissected on ice and cut into regions R0 to R5. Regionalization of the gut was determined according to Buchon et al. (59). Samples of with each gut region were homogenized in 100 µL of PBS 1× and split into equal parts (50 µL) for DNA extraction and RNA extraction. Gene expression and bacteria loads were determined by qPCR in paired samples.

### RNA Extraction and RT-qPCR.

RNA was extracted from 20 guts per treatment using TRIzol reagent as previously described (60). RNA concentration was determined by Nanodrop ND-1000 spectrophotometer. RT-PCR was performed using 500 ng of RNA in 10 µL volume of Solution with PrimeScript RT (TAKARA) and random hexamer primers. Quantitative PCR was performed in 384-well plates using the SYBR Select Master Mix from Applied Biosystems. Reads were performed on a LightCycler 480 (Roche).

### Samples Preparation for Proteomics.

Bacteria were cultured to OD 0.5. Cells were removed by centrifugation for 15 min at 3600 rpm. Supernatants were filtered through a 0.22-µm filter to remove any remaining bacteria. Twenty mL of supernatants was placed in Macrosep Advance Centrifugal Devices MWCO 3 kD (Pall Corporation) and centrifuged at max speed at 4 °C for 3 h. The concentrated supernatant solutions were transferred into Eppendorf tubes and sent to High Throughput Mass Spectrometry Core Facility (Charité) for proteomic analysis. The differential analysis of quantitative proteomics data was performed in Perseus v2.0.7.0.

### Isolation and Measurement of Peptidoglycan Acetylation.

*L. plantarum* PGN was extracted as described before (61). Measurement of peptidoglycan O-acetylation was performed as described previously (62) with certain modifications. Detailed procedures are explained in *SI Appendix*.

### Quantification of D-Alanylation of Teichoic Acids.

D-alanylation of teichoic acids was quantified as described previously (42, 63). Detailed procedures are explained in *SI Appendix*.

### Statistical Analysis.

Statistical parameters and tests are shown in the respective figure legends. Boxplots display boxes with the interquartile range from first to third quartiles; whiskers show the tenth and ninetieth percentiles. Statistical test was performed using R v4.2.2. Survival analysis and individual median survival were performed using Kaplan–Meier method and Log Rank test survival with the R package survminer. Comparisons were performed in pairs and plotted together. The R packages ggplot2, dplyr, and tidyverse were used for data visualization.

## Supplementary Material

Appendix 01 (PDF)

Dataset S01 (XLSX)

## Data Availability

16S rRNA gene amplicon sequencing data have been submitted to the NCBI database under BioProject no. PRJNA978012 (64). All study data are included in the article and/or supporting information.
