# Supplementary material for: Resistance to host antimicrobial peptides mediates resilience of gut commensals during infection and aging in Drosophila
Source: Proc Natl Acad Sci U S A. 2023 Aug 28;120(36):e2305649120. doi: 10.1073/pnas.2305649120 (PMC10483595; doi:10.1073/pnas.2305649120)
Supplement: Supplementary file 1 — Appendix 01 (PDF) [file pnas.2305649120.sapp.pdf]

Supporting Information for

Resistance to host antimicrobial peptides mediates resilience of gut commensals during infection and aging in *Drosophila*

Aranzazu Arias-Rojas[1], Dagmar Frahm[1], Robert Hurwitz[2], Volker Brinkmann[3], Igor Iatsenko[1*]

[1]Research group Genetics of host-microbe interactions, Max Planck Institute for Infection Biology, Charitéplatz 1, 10117 Berlin, Germany

[2] Protein Purification Core Facility, Max Planck Institute for Infection Biology

[3] Microscopy Core Facility, Max Planck Institute for Infection Biology

* **Corresponding author:** Igor Iatsenko

Email: iatsenko@mpiib-berlin.mpg.de

This PDF file includes:

SI Materials and Methods

Figures S1 to S4

Tables S1 to S2

Legends for Datasets S1

SI References

Other supporting materials for this manuscript include the following:

Datasets S1

SI Materials and Methods

Bacterial strains and culture conditions

The strains used in this study are listed in Table S1. *Escherichia coli* strains and *P. aeruginosa* were grown at 37 °C in LB medium with agitation. *L. plantarum* strains were grown in static conditions in MRS medium at 37 °C, unless stated otherwise. *Ecc15* and *P. entomophila* were grown at 30 °C in LB medium with agitation. *E. faecalis* was grown at 37 °C in BHI medium with agitation. *A. pomorum* was grown at 30 °C in MRS medium with agitation. *A. malorum, Sphingomonas sp, Variovorax sp, G. morbifer* were grown at 30 °C in mannitol medium with agitation. Erythromycin antibiotic was used at 5 μg/ml for *L. plantarum* and 150 μg/ml for *E. coli*. Chloramphenicol was used at 10 μg/ml. Solidified media were used for estimation of colony forming units (CFUs).

Generation of germ-free flies

To obtain axenic fly stocks, embryos laid over a 16-h period on grape juice plates were collected from 4- to 10-day-old females. Embryos were rinsed in phosphate-buffered saline (PBS) and transferred to 1.5 ml tubes. All following steps were performed in a sterile hood. Embryos were placed in a 3% solution of sodium hypochlorite for 10 min. The bleach solution was discarded and embryos were rinsed three times in sterile PBS. Embryos were transferred by pipette to tubes with antibiotics-supplemented food in a small amount of 100% ethanol and maintained at 25 °C. Subsequent generations were maintained in parallel to their conventionally reared counterparts by transferring adults to new tubes with antibiotics-supplemented food. The axenic state of flies was routinely assessed by culturing.

Generation and screening of random transposon mutant library in *L. plantarum* NCIMB8826

L. plantarum transposon mutagenesis was performed using the P_{junc}-TpaseIS*1223* system. Briefly, electrocompetent *L. plantarum* NCIMB8826 cells were first transformed with pVI129, resulting in the NCIMBpVI129 strain. Electrocompetent cells of the NCIMBpVI129 strain were transformed with pVI110, plated on MRS plates supplemented with 5 µg/ml of erythromycin and incubated for 48 h at 42 °C to select for integrants. A total of 3000 mutants were individually stored at -80 °C in deep 96-well plates. The whole transposon library was screened in 96-well plates for mutants not able to grow in MRS supplemented with 50 µg/ml of polymyxin B.

***Drosophila* mono-colonization-infection, priming-colonization, mono-colonization and infection in overexpression**

(a) Mono-colonization-infection: 10d old germ-free flies were starved for 2h prior to mono-colonization with *L. plantarum* or *A. malorum*. Overnight bacterial cultures were adjusted to OD=50 and mixed 1:1 with 5% sucrose. 150 µl of the mixture was placed on paper filter disks covering fly food and incubated at 25°C. After 48h of colonization, flies were starved for 2h at 29°C. After starvation, flies were infected with 150 µl of either alive or heat-killed *Ecc15*, OD=200 mixed 1:1 with 5% sucrose; control flies were treated only with 2.5% sucrose. Infection was performed at 29°C. 5 flies per treatment were used to record bacteria loads in MRS, Mannitol, or by qPCR, using total DNA at 6h and 24h post-infection. **(b)** Priming-colonization: 10d old germ-free flies were starved for 2h and primed with 150 µl of either alive or heat-killed *Ecc15* (OD200) at 29°C; control flies were treated only with sucrose 2.5%. After 3h of priming, flies were starved for 2h and mono-colonized with *L. plantarum* and *A. malorum* or infected with *Ecc15* or *Pe*, OD=200 mixed 1:1 with 5% sucrose. Control flies were treated only with 2.5% sucrose. Bacteria loads were recorded at 8h and 24h after priming using 5 flies per treatment. Bacteria loads were determined in MRS or Mannitol for microbiota and LB for pathogens or by qPCR, using total DNA. **(c)** mono-colonization and infection in

overexpression: Before colonization or infection, flies were maintained and starved for 2h at 29°C. 150 µl of *L. plantarum* or *A. malorum* OD=50 mixed 1:1 with 5% sucrose was added to a paper filter disk. For infection, 150 µl of *Ecc15* OD=200 mixed 1:1 with 5% sucrose was used. Bacteria loads were recorded by CFUs or qPCR at 6h, 24h, 48h, and 72h. Flies were flipped into conventional vials 48h post-treatment.

Aging and Survival

3d-old female flies were mono-colonized either with *L. plantarum* NC8 or *L. plantarum Δdltop,* following the same procedure as described above for mono-colonization. *L. plantarum* was given to flies only once at the beginning of the experiment. Bacterial loads were recorded by qPCR. 20 guts per sample were dissected to record the gene expression. Survival was recorded every 2 days and flies were flipped into new conventional vials every counting day.

Imaging and Immunostaining

DptA-GFP germ-free flies were mono-colonized with *L. plantarum* WJL-mCherry for 24h and infected with *Ecc15*. After 6h guts were fixed in PBS 1X with 4% paraformaldehyde. DNA was stained with DAPI 5 mg/ml (Sigma-Aldrich) 1:1000 in 1ml PBS overnight at 4°C, rinsed once with PBS 1X, and mounted in a chamber with Mowiol 4% (Sigma-Aldrich). Images were obtained using PS8 lightning confocal microscope. PH3 staining: 25d and 50d old guts were incubated in PBT 0.1% Triton X-100 + 0.3% BSA and 1:500 anti-PH3 mouse mAb IgG1 overnight at 4°C, rinsed in PBT and stained with secondary antibody Alexa555-coupled goat anti-rabbit antibody 1:500 and DAPI 5 mg/ml, 1:1000 at RT 1h in the Belydancer, rinsed in PBS and mounted in a chamber with Mowiol 4%. Images were obtained using a Leica DMR Fluorescence microscope. Anti-PH3 positive cells were measured through the fluorescence signal of Alexa555 using ImageJ. Complete guts area was selected to obtain the Mean Fluorescence Intensity (MFI) per gut among treatments.

Minimal Inhibitory Concentration (MIC) and growth kinetics

Overnight bacterial cultures were adjusted to OD 0.1, diluted 1:100, and pipetted (50 µl) into wells of 96 well plates prefilled with ranging dilutions of Polymyxin B (Fischer Scientific) or Cecropin B (Sigma). After overnight incubation, the bacterial growth was recorded to determine MIC value. To determine the kinetics of bacterial growth in presence of AMP *in vitro,* overnight cultures were adjusted to OD 0.05 and grew 20h in 96 well plates in a plate reader at 37°C in MRS medium supplemented with Polymyxin B. OD600 was measured. Reads were performed in Infinite 200 Pro plate reader (Tecan).

Cytochrome C and 5-FAM-LC-LL37 binding

Bacterial cultures were grown to OD 0.6 and resuspended in Buffer A (KH2PO4 1M pH: 7.0, BSA 0.01%) and Cytochrome C (Sigma) solution (0.5 mg/ml), the cells were incubated 15 min RT. Samples were centrifugated, and supernatants were measured in 96 well plates at 440 nm. Overnight cultures were adjusted to OD 0.1 in PBS 1X, and 5-FAM-LC-LL37 (Eurogentec) solution (14 µM) was added to each sample and incubated for 1h at 37°C, 590rpm. Samples were centrifugated for 1 min at max speed and supernatants were transferred to 96 well plates. Fluorescence was measured at absorbance 494 nm and 521 nm of emission. Reads were performed in Infinite 200 Pro plate reader (Tecan).

Mapping of transposon insertion sites

Genomic DNA from transposon mutants was digested sequentially with ClaI and BstBI restriction enzymes (NEB). These enzymes create compatible sticky ends facilitating ligation. Digested fragments were ligated using T4 DNA ligase (Thermo Fisher) according to the manufacturer's instructions. The resulting ligation products were transformed into the *E. coli* TG1 strain, in which circularized fragments that contain the transposon replicate as plasmids. Plasmids were isolated from selected transformants using Monarch plasmid miniprep kit (NEB)

and subjected to sequencing reactions (Eurofins) using the primers IRR6 and IRL6, which target the transposon sequence extremities. Identification of transposon target sequences was performed with the BLAST software from the National Center for Biotechnology Information (http://blast.ncbi.nlm.nih.gov/). Primers used in this study are listed in Table S2.

DNA extraction and 16S rRNA analysis

10d old w^{1118} iso conventional flies were infected with alive or heat-killed *Ecc15*, OD=200 mixed 1:1 with 5% sucrose. 150 µl of the mixture was applied to a filter disk covering the food surface of the vial with food; control flies were supplemented with 2.5% sucrose. Flies were incubated at 29 °C for 6h and 24h. 20 guts per sample were collected, and the total DNA was extracted using Puregene kit (Qiagen). Briefly, the guts were smashed in 300 µl of cell lysis solution with a pestle, in 1.5 ml Eppendorf tubes, and incubated 15 min at 65 °C. 100 µl of protein precipitation solution was added to each sample, vortexed, and incubated for 5 min on ice. Samples were spun down for 5 min at max speed; supernatants were transferred into new 1.5 ml Eppendorf tubes with 300 µl of cold isopropanol and pelleted at max speed for 10 min. Pellet was washed with 70% ethanol and spun down for 1 min at max speed. The dried pellet was dissolved in 20 µl of DNA hydration buffer. V3-V4 region of 16S rRNA gene was amplified using specific primers with the barcode and Phusion High-Fidelity PCR Master Mix (New England Biolabs). PCR products were mixed at equal density ratios. The mixed PCR products were purified with Qiagen Gel Extraction Kit (Qiagen, Germany). The libraries were generated with NEBNext UltraTM DNA Library Prep Kit for Illumina and quantified via Qubit and qPCR. The libraries were sequenced on NovaSeq 6000 system in paired end mode (Novogene). Analysis was performed using QIIME (1). 16S rRNA gene amplicon sequencing data have been submitted to the NCBI database under BioProject no. PRJNA978012.

Isolation and measurement of peptidoglycan acetylation

L. plantarum PGN was extracted as described before (2). Briefly, *L. plantarum* cells were grown in MRS medium and harvested by centrifugation at mid-exponential phase. Collected cells were adjusted to OD 10 and 2 ml of this cell suspension were transferred to 2 ml centrifuge tube. The cells were collected by centrifugation, washed with MilliQ water, boiled for 10 min and collected by centrifugation again. The pellet was resuspended in 1 ml of 5% SDS in 50 mM of MES (Sigma-M8250) pH 5.5, and boiled for 25 min at 100°C in a heating block. The pellets were collected by centrifugation, resuspended in 1 ml of 5% SDS in 50 mM of MES (Sigma-M8250) pH 5.5, and boiled for 15 min and then washed twice with MilliQ water to remove SDS traces. Next, the pellets were sequentially enzymatically treated with 2 mg/ml of Pronase (Roche 165921) in 50 mM of MES (Sigma-M8250) at pH 6.0 for 90 min at 60 °C; 200 µg/ml of trypsin (Sigma T-0303) in 50 mM of MES (Sigma-M8250) at pH 6.0 for 2 h at 37 °C with shaking; DNase (Sigma-D-4527) and RNase (Sigma-R-5503) (50 µg/ml) in 50 mM of MES (Sigma-M8250) at pH 6.0 for 1 h at 37 °C. After centrifugation, the pellets were resuspended in 2% SDS in 50 mM of MES (Sigma-M8250) at pH 5.5 and boiled for 15 min. Finally, the pellets were washed with MilliQ water to remove SDS traces, lyophilized and stored at -20 °C. Measurement of Peptidoglycan O-Acetylation was performed as described previously (3) with certain modifications. Briefly, lyophilized cell wall extracts (the same amount by weight per sample) were resuspended with 500 µl of 500 mM NaOH and incubated at room temperature for 30 minutes to release any ester-linked acetate. Solutions were neutralized with 500 mM H_2SO_4 and subjected to centrifugation. Quantification of released acetate was performed using Acetate Colorimetric Assay Kit (MAK086, Sigma), following manufacturer's instructions.

Scanning electron microscopy

Bacterial cells were fixed with 2.5% glutaraldehyde and 20 µl drops of bacterial suspension were spotted onto polylysine-coated round glass coverslips placed into the cavities of a 24-well cell culture plate. After 1 h of incubation in a moist chamber, PBS was added to the

wells, and the samples were fixed with 2.5% glutaraldehyde for 30 min. Samples were washed and post-fixed using repeated incubations with 1% osmium tetroxide and 1% tannic acid, dehydrated with a graded ethanol series, critical point dried and coated with 3 nm platinum/carbon. Specimens were analyzed in a Leo 1550 field emission scanning electron microscope using the in-lens detector at 20 kV. For quantification, images were recorded at a magnification of 2000 x and analyzed with the Volocity 6.5.1 software package.

Quantification of D-alanylation of teichoic acids

D-alanylation of teichoic acids was quantified following this protocol. Bacterial overnight cultures were centrifuged and resuspended in 1 ml of 1X PBS, washed with 1 ml of acetate ammonium (20 mM pH: 4.7). Solution was split into 1.5 ml Eppendorf tubes at OD 50 and heat-inactivated at 100 °C for 10 min. Cells were frozen with nitrogen stream for 1 min and lyophilized in the vacuum chamber overnight. d-alanine was released from heat-inactivated bacteria by mild alkaline hydrolysis with 0.1 N NaOH for 1 h at 37 °C. Finally, the solutions were neutralized with 0.1 N HCl. After neutralization, the extracts were incubated with Marfey's reagent (1-fluoro-2,4-dinitrophenyl-5-L-alanine amide; Sigma). After drying in a nitrogen stream, the residues were derivatized with 50 µL of 0.5% Marfey's reagent (w/v in acetone) and 100 µL of 125 mM disodiumtetraborate for 30 min at 40 °C. The reaction was stopped with 25 µL pf 4 M HCl. The resulting solution was diluted (1:10) with 4 mM ammonium formiate, pH 4.6). Two µL of the derivatized samples were subjected on an Acquity H-Class UPLC system (Waters), using an AccQ-Tag Ultra C18 column (1.7µm, 2.1x100mm), and a linear gradient of 100% mobile phase A (4mM ammonium formiate; pH 4.6) to 50% mobile phase A and B (acetonitrile) within 5 min at 0.5 mL/min. Derivatized amino acids were detected both, by UV-absorbance (340 nm) and by mass spectrometry (negative ion mode at m/z 340) with a single quadrupole mass spectrometer (QDA, Waters). For quantification, standard D-alanine was analysed at 5 concentrations (0-100µM) which were prepared in duplicate in 0.1 N HCl before derivatization.

Supplementary figures

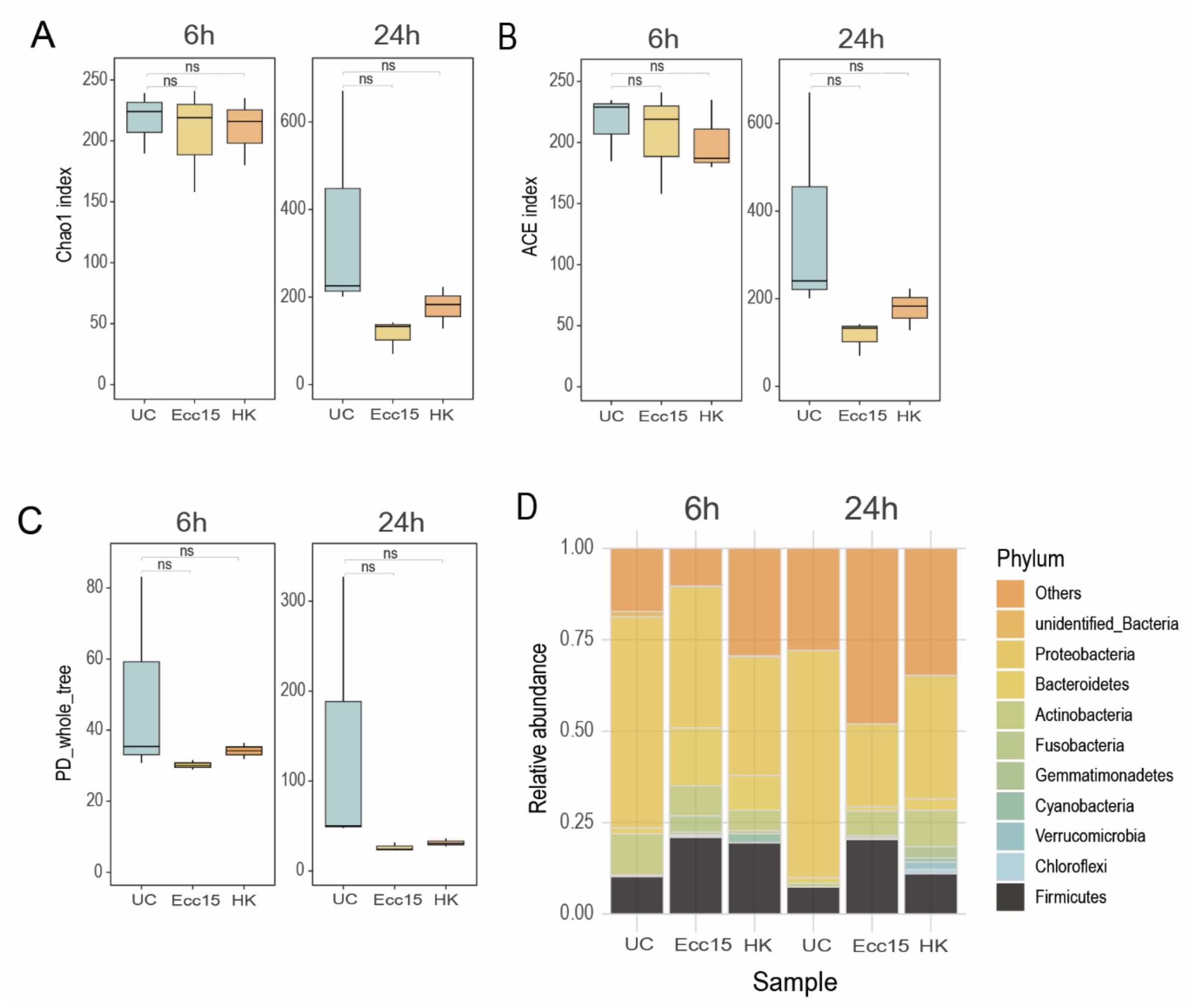

Figure S1. Abundance of *Drosophila* microbiota communities during infection. Alpha diversity indexes illustrated by Chao1 (*A*), ACE (*B*), and PD whole tree index (*C*) 6h and 24h after the infection with *Ecc15* alive and heat-inactivated in 10d old conventional files (n=3 independent experiments with 20 guts per treatment and time point). (*D*) The relative abundance of 10 dominant OTUs after *Ecc15* infection at the Phylum level. Boxplots show median and interquartile range (IQR), and whiskers show either the lower and upper quartiles or range. *P < 0.05, **P < 0.01, ***P < 0.001, ****P < 0.0001. Kruskal–Wallis and Bonferroni post hoc tests were used for statistical analysis.

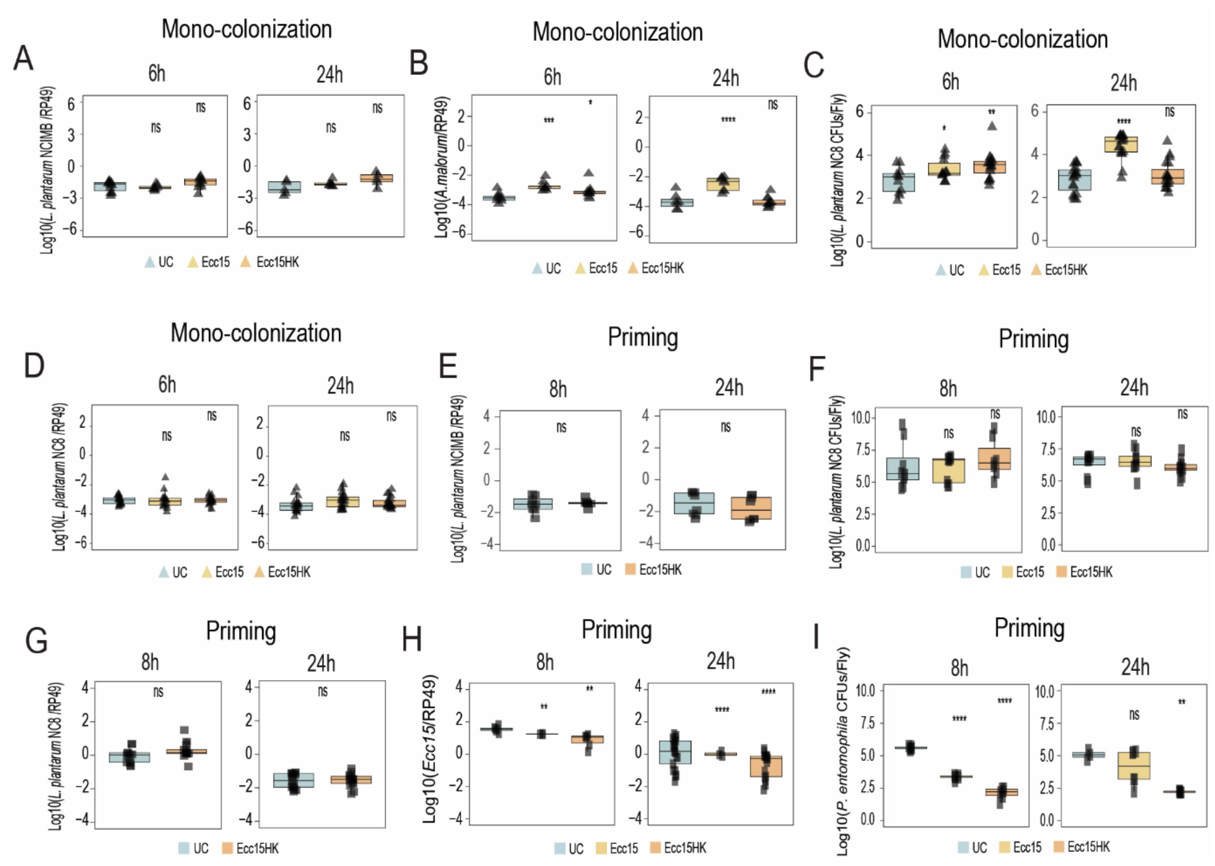

Figure S2. Status of single *Drosophila* microbiota members and pathogens during infection, priming, and overexpression of IMD pathway in *Drosophila* gut. Mono-colonization-Infection: (*A*) *L. plantarum*^{NCIMB} loads measured by qPCR after the infection with *Ecc15*, (*B*) *A. malorum* loads measured by qPCR after the infection with *Ecc15*. (a-b, n=8 independent samples per treatment with 5 flies per sample). (*C-D*) *L. plantarum*^{NC8} loads measured by CFUs (*C*) and qPCR (*D*) at 6h and 24h after the infection with *Ecc15*. The single triangles are mean bacterial load values from pools of n = 5 animals in the Log10 scale. **Priming:** (*E*) *L. plantarum*^{NCIMB} loads measured by qPCR after the priming with *Ecc15*. (*F-G*) *L. plantarum*^{NC8} loads measured by CFUs (*F*) and qPCR (*G*) at 6h and 24h after the priming with *Ecc15*. (*H*) *Ecc15* loads measured by qPCR after the priming with *Ecc15* (*I*) *P. entomophila* loads measured by CFUs after the priming with *Ecc*15. (f-k, n=8 independent samples per treatment with 5 flies per sample). The single squares are mean bacterial load values from pools of n = 5 animals in the Boxplots and dot plots show median and interquartile ranges (IQR); whiskers show either lower or upper quartiles or ranges. *P < 0.05, **P < 0.01, ***P < 0.001, ****P < 0.0001. Kruskal–Wallis and Bonferroni post hoc tests were used for statistical analysis.

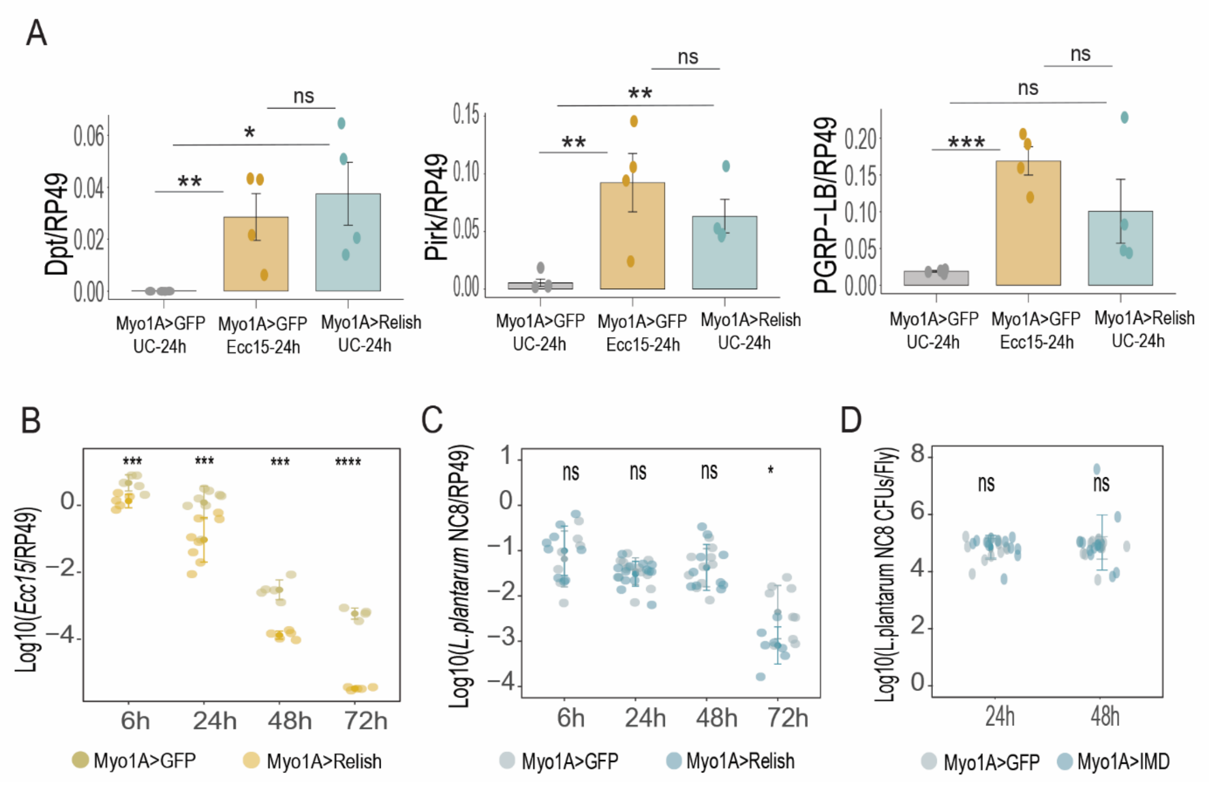

Figure S3. Genetic activation of IMD pathway triggers AMP response in the gut without affecting *L. plantarum* load. (*A*) Expression of *DptA*, *Pirk1*, and *PGRP-LB* in *Myo1A-GAL4>UAS-GFP* uninfected (UC) (n=4) and infected with alive Ecc15 (n=4) and in uninfected *Myo-GAL4>UAS-Relish* flies (n=4). 20 guts per sample were dissected for gene expression. (*B-C*) *Ecc15* (*B*) and *L. plantarum*^{NC8} (*C*) loads in *Myo1A-GAL4>UAS-GFP* and *Myo-GAL4>UAS-Relish* flies at 6h, 24h, 48h, and 72h after colonization or infection (n=10 per time point and genotype). (*D*) *L. plantarum*^{NC8} loads in *Myo1A-GAL4>UAS-GFP* and *Myo-GAL4>UAS-Imd* flies at 24h and 48h after colonization (n=10 per time point and genotype). Boxplots and dot plots show median and interquartile ranges (IQR); whiskers show either lower or upper quartiles or ranges. *$P < 0.05$, **$P < 0.01$, ***$P < 0.001$, ****$P < 0.0001$. Kruskal–Wallis and Bonferroni post hoc tests were used for statistical analysis.

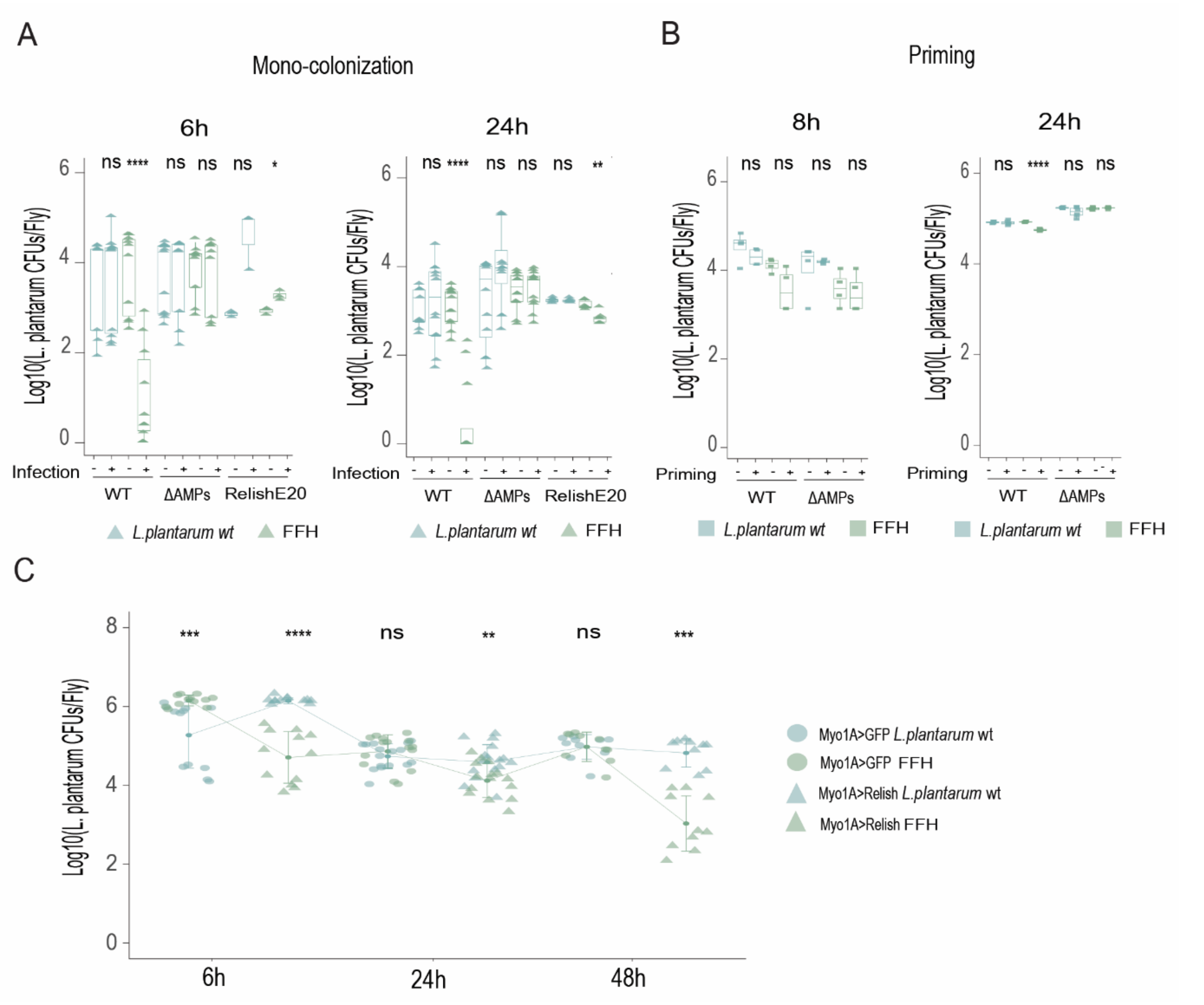

Figure S4. *L. plantarum ffh* gene is essential to colonize and persist in the gut during infection. (*A*) *L. plantarum*^{NCIMB} and *L. plantarum*^{ffh} loads in wild type (n=10 per treatment), Δ*AMPs* (n=10 per treatment) and *Relish*^{E20} (n=3 per treatment) flies after the infection with *Ecc15* at 6h and 24h. (*B*) *L. plantarum*^{NCIMB} and *L. plantarum*^{ffh} loads after the priming with *Ecc15* at 8h and 24h in wild type and Δ*AMPs* (n=9 per treatment). (*C*) *L. plantarum*^{NCIMB} and *L. plantarum*^{ffh} loads in *Myo1A-GAL4>UAS-GFP* and *Myo-GAL4>UAS-Relish* at 6h (n=12 per treatment), 24h (n=16 per treatment), and 48h (n=10 per treatment) after colonization. Individual tringles, squares and dots show mean CFU values from pools of n = 5 animals in the Log10 scale. Boxplots and dot plots show median and interquartile ranges (IQR); whiskers show either lower or upper quartiles or ranges. *P < 0.05, **P < 0.01, ***P < 0.001, ****P < 0.0001. Kruskal–Wallis and Bonferroni post hoc tests were used for statistical analysis.

Supplementary tables

Table S1. Bacterial strains and plasmids used in this study.

Strain or plasmid	Relevant characteristics	Reference or source
**Strains**		
Pectobacterium carotovorum carotovorum (Ecc15)	Natural *Drosophila* pathogen	(4)
Pseudomonas entomophila	Lethal intestinal pathogen of *Drosophila*	(5)
Pseudomonas aeruginosa PA14	Pathogen used for *Drosophila* intestinal infection	(6)
Levilactobacillus brevis DSM20556		German Collection of Microorganisms and Cell Cultures (DSMZ)
Acetobacter pomorum WJL	*Drosophila* isolate	(7)
Acetobacter malorum DSM14337		German Collection of Microorganisms and Cell Cultures (DSMZ)
Sphingomonas leidyi	*Drosophila* isolate	This study
Variovorax spp	*Drosophila* isolate	This study
Gluconobacter morbifer G707	*Drosophila* isolate	(8)
Entorococcus faecalis	*Drosophila* commensal	(9)
Lactiplantibacillus plantarum NCIMB 8826 (WCFS1)	Strain with high transformation efficiency	(10)
L. plantarum NC8		(11)
L. plantarum WJL	*Drosophila* isolate	(8)
L. plantarum WJL-mCherry		(12)
Δ*dltop*	NC8 strain deleted for *pbpX2* and *dltXABCD* genes	(11)
L. plantarum P17E3	NCIMB strain with transposon insertion in *dlt* operon	This study
L. plantarum P21C9	NCIMB strain with transposon insertion in *ffh* gene	This study
L. plantarum NCIMBpVI129	NCIMB strain carrying pVI129	This study
L. plantarum NZ7100	Derivative of WCFS1	(13)

L. plantarum OatA-	NZ7100 oatA ::lox72, strain lacking *OatA*	(13)
L. plantarum OatB-	NZ7100 oatB ::lox72, strain lacking *OatB*	(13)
L. plantarum OatAB-	Strain lacking *OatA* and *OatB*	(13)
L. plantarum P21C9pGIEB003	P21C9 mutant containing pGIEB003 plasmid for overexpression of OatA	This study
L. plantarum P21C9 pNZ8048::ffh	P21C9 mutant containing pNZ8048::ffh plasmid for ffh overexpression	This study
E. coli TG1	supE hsd5h thi (Δlac-proAB) F' (traD36 proAB-lacZΔM15)	(14)
**Plasmids**		
pVI129	Ap^r Cm^r, pVI1056 containing Phlb A-IS1223ΔIR	(15)
pVI110	Em^r, pBR322ori, Pjunc	(15)
pNZ8048	Cm^r; shuttle vector containing PnisA promoter and start codon in NcoI site	(16)
pGIEB003	Cm^r; pNZ8048 derivative containing *oatA* gene in transcriptional fusion	(13)
pNZ8048::ffh	Cm^r; pNZ8048 derivative containing *ffh* gene	This study

Table S2. Primers used in this study.

Primer name	Sequence 5′→3′	Source
IRR6	TCACCGTCATCACCGAAACG	(15)
IRL6	GCCGCACTAGTGATTAAAATAC	(15)
L. plantarum Fwd	TGGAAACAGATGCTAATACCG	This study
L. plantarum Rev	GTCCATTGTGGAAGATTCCC	This study
A. malorum Fwd	TTGACCTTAAGCCGGTGAGC	This study
A. malorum Rev	TCCCCTACGGCTACCTTGTT	This study
Ecc15 Fwd	GCCGATAGCCCATCAACAGA	This study
Ecc15 Rev	CCTTATGCCTATCGGAGGCG	This study
RP49F	GACGCTTCAAGGGACAGTATCTG	(17)
RP49R	AAACGCGGTTCTGCATGAG	(17)
DptA F	GCTGCGCAATCGCTTCTACT	(17)
DptA R	TGGTGGAGTGGGCTTCATG	(17)
Socs36E F	CACAGCAGCAAGCCAGTTTC	This study
Socs36E R	AGTGCTTTACTGCTGCGACT	This study
Upd3 F	GCGGGGAGGATGTACC	(17)
Upd3 R	GTCTTCATGGAATGAGCC	(17)

Dataset S1 (separate file). Proteomic analysis of secreted and surface-associated proteins in *L. plantarum NCIMB* and *ffh* mutant

SI References

1. J. G. Caporaso, *et al.*, QIIME allows analysis of high-throughput community sequencing data. *Nat. Methods 2010 75* **7**, 335–336 (2010).
2. D. Kühner, M. Stahl, D. D. Demircioglu, U. Bertsche, From cells to muropeptide structures in 24 h: Peptidoglycan mapping by UPLC-MS. *Sci. Reports 2014 41* **4**, 1–7 (2014).
3. M. H. Laaberki, J. Pfeffer, A. J. Clarke, J. Dworkin, O-Acetylation of Peptidoglycan Is Required for Proper Cell Separation and S-layer Anchoring in Bacillus anthracis. *J. Biol. Chem.* **286**, 5278 (2011).
4. A. Basset, *et al.*, The phytopathogenic bacteria Erwinia carotovora infects Drosophila and activates an immune response. *Proc. Natl. Acad. Sci.* **97**, 3376–3381 (2000).
5. N. Vodovar, *et al.*, Drosophila host defense after oral infection by an entomopathogenic Pseudomonas species. *Proc. Natl. Acad. Sci. U. S. A.* **102**, 11414–9 (2005).
6. L. G. Rahme, *et al.*, Common virulence factors for bacterial pathogenicity in plants and animals. *Science (80-.).* **268**, 1899–1902 (1995).
7. S. C. Shin, *et al.*, Drosophila microbiome modulates host developmental and metabolic homeostasis via insulin signaling. *Science (80-.).* **334**, 670–674 (2011).
8. J.-H. Ryu, *et al.*, Innate immune homeostasis by the homeobox gene caudal and commensal-gut mutualism in Drosophila. *Science* **319**, 777–82 (2008).
9. A. Marra, M. A. Hanson, S. Kondo, B. Erkosar, B. Lemaitre, *Drosophila* Antimicrobial Peptides and Lysozymes Regulate Gut Microbiota Composition and Abundance. *MBio* (2021) https:/doi.org/10.1128/mBio.00824-21
10. M. Kleerebezem, *et al.*, Complete genome sequence of Lactobacillus plantarum WCFS1. *Proc. Natl. Acad. Sci. U. S. A.* **100**, 1990–1995 (2003).
11. R. C. Matos, *et al.*, D-Alanylation of teichoic acids contributes to Lactobacillus plantarum-mediated Drosophila growth during chronic undernutrition. *Nat. Microbiol.* **2**, 1635–1647 (2017).
12. G. Storelli, *et al.*, Drosophila Perpetuates Nutritional Mutualism by Promoting the Fitness of Its Intestinal Symbiont Lactobacillus plantarum. *Cell Metab.* **27**, 362-377.e8 (2018).
13. E. Bernard, *et al.*, Characterization of O-Acetylation of N-Acetylglucosamine: A NOVEL STRUCTURAL VARIATION OF BACTERIAL PEPTIDOGLYCAN. *J. Biol. Chem.* **286**, 23950–23958 (2011).
14. T. Clackson, H. R. Hoogenboom, A. D. Griffiths, G. Winter, Making antibody fragments using phage display libraries. *Nature* **352**, 624–628 (1991).
15. H. Licandro-Seraut, *et al.*, Development of an efficient In vivo system (Pjunc-TpaseIS1223) for random transposon mutagenesis of Lactobacillus casei. *Appl. Environ. Microbiol.* **78**, 5417–5423 (2012).
16. O. P. Kuipers, P. G. G. A. De Ruyter, M. Kleerebezem, W. M. De Vos, Quorum sensing-controlled gene expression in lactic acid bacteria. *J. Biotechnol.* **64**, 15–21 (1998).

17. I. Iatsenko, J.-P. Boquete, B. Lemaitre, Microbiota-Derived Lactate Activates Production of Reactive Oxygen Species by the Intestinal NADPH Oxidase Nox and Shortens Drosophila Lifespan. *Immunity* **49**, 929-942.e5 (2018).
